# Synergistic ROS Generation via Core–Shell Nanostructures with Increased Lattice Microstrain Combined with Single-Atom Catalysis for Enhanced Tumor Suppression

**DOI:** 10.1021/acsami.4c10392

**Published:** 2024-08-15

**Authors:** Liu-Chun Wang, Li-Chan Chang, Hsiang-Lin Huang, Po-Ya Chang, Chih-Wen Pao, Yin-Fen Liu, Keng-Shiang Huang, Yi-Hsin Chien, Hwo-Shuenn Sheu, Wen-Pin Su, Chen-Hao Yeh, Chen-Sheng Yeh

**Affiliations:** † Department of Chemistry, National Cheng Kung University, Tainan 701, Taiwan; ‡ Center of Applied Nanomedicine, National Cheng Kung University, Tainan 701, Taiwan; § Institute of Clinical Medicine, College of Medicine, National Cheng Kung University, Tainan 704, Taiwan; ∥ National Synchrotron Radiation Research Center, Hsinchu 30076, Taiwan; ⊥ The School of Chinese Medicine for Post-Baccalaureate, I-Shou University, Kaohsiung City 82445, Taiwan; # Department of Materials Science and Engineering, Feng Chia University, Taichung 40724, Taiwan; ∇ Departments of Oncology and Internal Medicine, National Cheng Kung University Hospital, College of Medicine, National Cheng Kung University, Tainan 704, Taiwan; ○ Clinical Medicine Research Center, National Cheng Kung University Hospital, College of Medicine, National Cheng Kung University, Tainan 704, Taiwan

**Keywords:** core−shell effect, single-atom catalyst, strain effect, Fenton-like reaction, chemodynamic therapy

## Abstract

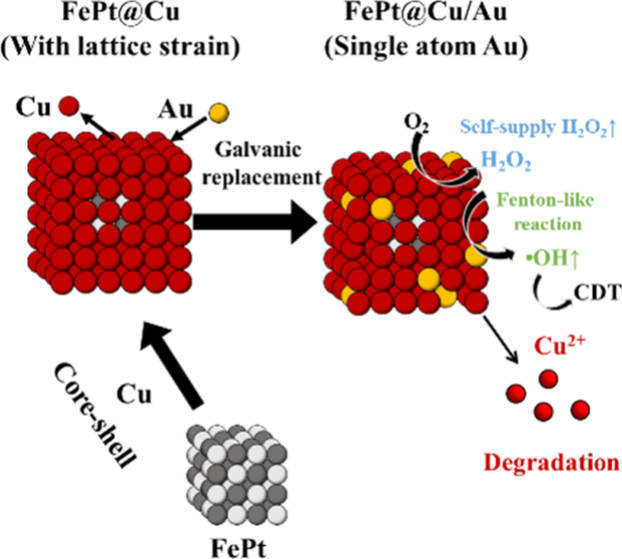

This study emphasizes the innovative application of FePt and Cu core–shell nanostructures with increased lattice microstrain, coupled with Au single-atom catalysis, in significantly enhancing ^•^OH generation for catalytic tumor therapy. The combination of core–shell with increased lattice microstrain and single-atom structures introduces an unexpected boost in hydroxyl radical (^•^OH) production, representing a pivotal advancement in strategies for enhancing reactive oxygen species. The creation of a core–shell structure, FePt@Cu, showcases a synergistic effect in ^•^OH generation that surpasses the combined effects of FePt and Cu individually. Incorporating atomic Au with FePt@Cu/Au further enhances ^•^OH production. Both FePt@Cu and FePt@Cu/Au structures boost the O_2_ → H_2_O_2_ → ^•^OH reaction pathway and catalyze Fenton-like reactions. This enhancement is underpinned by DFT theoretical calculations revealing a reduced O_2_ adsorption energy and energy barrier, facilitated by lattice mismatch and the unique catalytic activity of single-atom Au. Notably, the FePt@Cu/Au structure demonstrates remarkable efficacy in tumor suppression and exhibits biodegradable properties, allowing for rapid excretion from the body. This dual attribute underscores its potential as a highly effective and safe cancer therapeutic agent.

## 1 Introduction

Catalytic nanomaterials with enzyme-mimicking activities have garnered significant research attention in recent years, particularly in the context of various diseases including malignant tumors. These nanomaterials are capable of catalyzing H_2_O_2_ or O_2_ to generate toxic reactive oxygen species (ROS) products, such as hydroxyl radicals (^•^OH), superoxide anions (O_2_^–^), and singlet oxygen (^1^O_2_), thereby inducing apoptosis and damaging tumor cells. Consequently, cancer therapeutic strategies activated by the tumor microenvironment have been widely developed to target lesions. However, the limited endogenous presence of H_2_O_2_ or O_2_ in tumors following tumor-microenvironment-activated approaches has significantly compromised therapeutic efficacy. For instance, the intratumoral H_2_O_2_ levels typically remain below approximately 100 μM due to cellular redox homeostasis, which cannot effectively provide a sustained supply of H_2_O_2_.

One promising approach is to advance nanocatalysis to the atomic level by creating single-atom catalysts (SACs).^1,2^ The atomically dispersed metal structure demonstrates efficient metal utilization, establishing catalytic sites that offer significant advantages for designing novel nanocatalysts.^3^ Combining highly reactive atoms with catalytic activity has endowed SACs with the ability to achieve synergistic therapeutic outcomes with minimized side effects against malignant tumors. Among the single-atom catalytic treatments, the generation of ^•^OH (via Fenton, Fenton-like, or peroxidase-like reactions),^4,5^ O_2_^–^ (through oxidase-like reactions),^6,7^ or ^1^O_2_ (via catalase-like reactions)^8^ has been explored against tumors. While SACs have proven effective in enhancing catalytic efficiency, it is essential to recognize that endogenous H_2_O_2_ or O_2_ remains a primary source for catalytic reactions and the production of ROS. Therefore, alternative approaches to overcome this endogenous limitation are highly desired to boost the availability of ROS for tumor catalytic treatments.

Several elegant strategies have been employed to elevate ROS levels in this context. For instance, in the case of SACs, the delicate design of formulations based on Cu, Fe, Mn, and Co^9,10^ can concurrently generate multiple ROS. Taking the concept of cocatalysis characterized by catalytic loop dynamics, an efficient supply of ^•^OH is achieved through the Fenton or Fenton-like reaction.^11,12^ For instance, this strategy is implemented using the reductive capability of active Mo^4+^ to Mo^6+^, thereby accelerating the conversion of Fe^3+^ to Fe^2+^.^13^ Apart from SACs, a similar scenario reveals an efficient catalytic loop in bimetallic such as CuFe nanocatalysts, where Cu^+^ → Cu^2+^-mediated conversion of Fe^3+^ → Fe^2+^ enhances ^•^OH generation along with self-supply of H_2_O_2_.^14^ From a structural perspective, the heterogeneous growth nanocatalysts, characterized by configurations such as core–shell structures and alloys, offer activity sites that boost the ^•^OH generation reaction, particularly in defect-rich or lattice-mismatched regions.^15,16^ In addition, the creation of a flower-like structure allows for 3D accessibility to active sites, boosting ^•^OH generation.^17^ Recently, we used galvanic replacement reactions to fabricate atomically dispersed Au on Cu nanocubes. This demonstrated the self-supply of H_2_O_2_ and the ability to generate H_2_O_2_ readily and ^•^OH from O_2_.^18^

Herein, we demonstrate that the formation of a core–shell structure with increased lattice microstrain surprisingly boosts ^•^OH generation, potentially adding a new dimension to the ROS enhancement in catalytic tumor treatments. We selected FePt and Cu to construct core–shell nanocubes due to their degradable properties, which yield metal ions under tumor acidic conditions.^19−21^ This combination was initially anticipated to result in a summation effect (1 + 1 = 2) in ^•^OH production through the Fenton reaction from Fe^2+^ (from degradable FePt) and Cu^+^ (from degradable Cu). Unexpectedly, we observed a synergy effect (1 + 1 > 2) in ^•^OH production. Core–shelled FePt@Cu nanocubes exhibited a significant enhancement compared to the sum of ^•^OH generated by individual FePt nanoparticles and Cu nanocubes. For instance, FePt@Cu nanocubes showed a 3.9-fold increase compared to Cu and a 42-fold increase compared to FePt nanoparticles in ^•^OH production. FePt@Cu nanocubes follow the reactions of the oxidizing oxidizers of O_2_→ H_2_O_2_→ ^•^OH. Theoretical calculations indicate a lower O_2_ adsorption energy in the core–shell structure. We suggest that the lattice mismatch resulting in strain effect^22^ between FePt and Cu has increased the activity of the Cu shell. Moreover, when we further fabricated core–shell FePt@Cu nanocubes using galvanic replacement to introduce atomic Au onto FePt@Cu, creating FePt@Cu/Au, ^•^OH production was further enhanced. For instance, FePt@Cu/Au exhibited a 4.8-fold increase compared with Cu and a 52-fold increase compared to FePt in ^•^OH production. This study systematically explores the synergistic effect of ^•^OH production from core–shell structures combined with single-atom structures. The group that combines the core–shell effect and the single-atom structure of FePt@Cu/Au demonstrated significantly improved tumor suppression with notable progress observed for 25 days post-treatment. Moreover, the FePt@Cu/Au compound has shown the ability to be excreted via urine within a day after injection in *in vivo* studies, a feature attributed to its biodegradable nature.

## 2 Experimental Section

### 2.1 Chemicals

All reagents were analytically pure and used without further purification. Ethanol (C_2_H_5_OH, 99.9%) was purchased from J. T. Baker. Aminophenyl fluorescein solution (APF, C_26_H_17_NO_5_, 98%) was acquired from Life Technologies. Hydrogen peroxide assay kit was acquired from abcam. CopperGreen dyes were obtained from Merck. Copper(I) bromide (CuBr, 98%), octadecylamine ((ODA), CH_3_(CH_2_)_17_NH_2_, 99%), trioctylphosphine oxide ((TOPO), [CH_3_(CH_2_)_7_]_3_PO, 90%), Oleylamine ((Oam), CH_3_(CH_2_)_7_CH=CH(CH_2_)_7_CH_2_NH_2_, 90%), cetyltrimethylammonium bromide (C_19_H_42_BrN), polyvinylpyrrolidone (PVP, (C_6_H_9_NO)n, M.W.= 55000), hydrogen peroxide solution (H_2_O_2_, 30%), sodium bromide (NaBr, 99.5%), ascorbic acid (C_6_H_8_O_6_, 99%), cetyltrimethylammonium chloride (C_19_H_42_ClN, 25%), sodium borohydride (NaBH_4_, 99%), and 3-(4,5-dimethylthiazol-2-yl)-2,5-diphenyltetrazolium bromide (MTT, C_18_H_16_BrN_5_S, 97.5%) were bought from Sigma-Aldrich. Water was obtained using a Millipore direct-Q deionized water system throughout all studies.

### 2.2 Cell Lines

Human hepatocellular carcinoma HepG2-Red-FLuc cells were cultured in minimum essential medium (MEM) containing 10% fetal bovine serum (FBS) and 100 U/mL penicillin-streptomycin at 37 °C with 5% CO_2_. HUV-EC-C cells (endothelial cell line) were cultured in F-12k containing EGCS (0.03 mg/mL), heparin (0.1 mg/mL), and fetal bovine serum (FBS, 10%) in an incubator at 37 °C and 5% CO_2_

### 2.3 Mice

The care of animals adhered to the Laboratory Animal Welfare Act and the Guidelines for the Care and Utilization of Laboratory Animals, receiving approval from the Institutional Animal Care and Use Committee (IACUC) at the National Cheng Kung University (NCKU). Every animal treatment and surgical procedure followed the protocols outlined by the NCKU Laboratory Animal Center (IACUC no. 112192). The experimental mice were kept in cages under conditions of 22–23 °C temperature and 55 ± 10% humidity, following a light/dark cycle of 13 h/11 h.

### 2.4 Preparation of FePt Nanoparticles

FePt nanoparticles were prepared by mixing the solution of 0.2 g of platinum acetylacetonate and 0.13 mL of Fe(CO)_5_ in 10 mL of octyl ether into a mixture of 10 mL of octyl ether that contained 0.39 g of 1,2-hexadecanediol, 0.17 mL of oleic acid, and 0.16 mL of oleylamine. Then, the reaction apparatus was filled with argon, heated at 10 °C/min in a heating jacket, and maintained at 250 °C for 1 h. The reaction solution was cooled to room temperature and centrifuged at 6200*g* for 5 min. The final product was collected by centrifuging and washing with ethanol and hexane. Finally, FePt nanoparticles were collected and stored in oleylamine.

### 2.5 Preparation of FePt@Cu Nanocubes

We mixed 0.02 g of CuBr, 0.08 g of octadecylamine, 1 g of trioctylphosphine oxide, and 100 μL of FePt nanoparticles (with an iron concentration fixed at 1000 ppm) in 20 mL of oleylamine. The mixture was then placed in a reaction setup, purged with argon, and heated at a rate of 20 °C/min within a heating jacket. The temperature was held at 300 °C for 10 min, facilitating the formation of FePt@Cu nanocubes. After the reaction, the mixture was allowed to cool to ambient temperature and then centrifuged at 6200*g* for 5 min. The supernatant was discarded, and the precipitate was washed three times with a toluene solution. The FePt@Cu nanocubes were ultimately retrieved and stored in oleylamine for further use.

### 2.6 Preparation of FePt@Cu/Au Nanocubes

In this investigation, solutions of cetyltrimethylammonium bromide (CTAB) and poly(vinylpyrrolidone) (PVP) facilitated the transfer of FePt@Cu nanocubes from the oil phase into the water phase. The FePt@Cu nanocubes then served as a sacrificial template in the galvanic replacement reaction, with the acidic HAuCl_4_ solution acting as the metal precursor. Initially, the FePt@Cu nanocubes were dispersed in a 100 μL toluene solution at a concentration of 10 000 ppm. Then, 10 mL of a CTAB and PVP solution was added and thoroughly mixed, emulsifying the FePt@Cu nanocubes from the oil phase into the water phase and ensuring their dispersion in the aqueous medium. To form FePt@Cu/Au nanocubes, 100 μL of a HAuCl_4_ solution with a molar concentration of 0.05 mM was promptly introduced.

### 2.7 Preparation of Cu Nanocubes

A blend of 0.05 g of CuBr, 0.08 g of octadecylamine, and 1 g of trioctylphosphine oxide was combined with 20 mL of oleylamine. This concoction was then saturated with argon within the experimental apparatus, heated at a rate of 20 °C/min using a heating jacket, and kept at 260 °C for 10 min, promoting the transformation of Cu nanoparticles into nanocubes. Following the reaction, the mixture was allowed to cool to room temperature and then centrifuged at 8000 rpm for 5 min. The supernatant was removed, and the nanocubes underwent three rounds of purification using toluene. The resulting Cu nanocubes were collected and preserved in oleylamine.

### 2.8 Preparation of Au Nanocubes

Initially, gold seeds were synthesized using a solution composed of 10 mL of water, 1325 μL of cetyltrimethylammonium chloride (CTAC) at a 25% concentration, 500 μL of a 5 mM HAuCl_4_ solution, and 450 μL of a 0.02 M NaBH_4_ solution. Following this, two separate vials, labeled A and B, were prepared for the growth process. In each vial, a growth mixture was created with 10 mL of water, 1325 μL of CTAC (25%), 500 μL of the 5 mM HAuCl_4_ solution, 10 μL of a 0.01 M NaBr solution, and 90 μL of a 0.04 M ascorbic acid solution. Subsequently, 25 μL of the prepared seed solution was introduced into the mixture in vial A and allowed to react for 10 min. After this period, 25 μL from vial A was transferred to vial B, where it was stirred for an additional 15 min. The resulting Au nanocubes were then harvested and stored in water.

### 2.9 Characterization of Crystal Structure

The crystal structure was analyzed by synchrotron X-ray diffraction (XRD) with the incident X-ray wavelength set to 16 keV (0.77491 Å). This analysis was conducted using Debye–Scherrer technology at beamline 01C2 of the Taiwan Light Source (TLS), located at the National Synchrotron Radiation Research Center (NSRRC). The electron storage ring was operated at 1.5 GeV and 362 mA under top-up injection. Powder XRD patterns were collected by using a transmission-type setup. The powder samples were sealed within two layers of Scotch tape in a glovebox under dry N_2_ atmospheres to prevent oxidation from air exposure. Two-dimensional powder X-ray diffraction patterns were recorded by using a mar345 imaging plate detector. Spatial geometry calibration was performed using the SRM 674b CeO_2_ powder as the standard. The 2D patterns were integrated to obtain 1D XRD patterns via GSAS-II software.^23^ The 1D XRD pattern was then deconvoluted to obtain the full width at half-maximum (fwhm) using the Origin program. Subsequently, the Williamson–Hall plot^24^ was employed to determine the crystalline grain size, enabling the calculation of microstrain for a series of nanocubes.

### 2.10 Characterization of Atomic Environment

X-ray absorption spectroscopy encompasses X-ray absorption near-edge spectra (XANES) and extended X-ray absorption fine structure (EXAFS). The experiments were carried out in transmission type for Cu K-edge, Pt and Au L_3_-edges at beamline TPS 44A of the Taiwan Photon Source (TPS). Since the fluorescence lines of Cu and Pt are close to the Au L_3_-edge and the loading of Cu is very high compared to Au and Pt, an energy-resolved fluorescence spectrometer utilizing seven-element Silicon Drift Detectors (SDD) was employed to detect the weak Au and Pt fluorescence signal at TPS 44A.^25^ Spectra were obtained by subtracting the baseline of the pre-edge and normalizing that of the post-edge using Athena software. EXAFS analysis involved Fourier transform of k^2^-weighted EXAFS oscillations to assess the contribution of each shell to the Fourier transform peak, followed by fitting using Artemis software.^26^

### 2.11 Computational Details

In this study, all periodic density functional theory (DFT) calculations were conducted utilizing the Perdew–Burke–Ernzerhof (PBE) exchange-correlation functional^27^ within the generalized gradient approximation (GGA) framework, employing the Vienna Ab initio Simulation Program (VASP).^28−31^ The projector-augmented wave (PAW) method^32,33^ was employed to describe electron–core interactions accurately. Kohn–Sham orbitals were expanded using a plane-wave basis set with a kinetic energy cutoff of 400 eV while spin polarization was considered. Convergence criteria were 1 × 10^–5^ eV for the total electronic energy within the self-consistent loop. Atomic positions were relaxed using the Conjugate Gradient method until the unconstrained atomic forces along the x-, y-, and z-components were smaller than 1 × 10^–2^ eV/Å.

Metal and core–shell systems, including pure Cu, FePt@Cu, and FePt@Cu/Au, were modeled by using the FCC cubic unit cell. The (111) slab model was employed for all systems, utilizing a (2 × 2) supercell for lattice mismatch analysis and a (3 × 3) supercell for surface reaction calculations. A vacuum spacing of 15 Å was implemented between the slab and its periodic replicas. Brillouin zone sampling was achieved using a Monkhorst–Pack mesh^34^ of (10 × 10 × 10) for unit cells and (5 × 5 × 1) for supercells of metal and core–shell systems. The calculated lattice constants for bulk FePt and Cu were found to be 3.85 and 3.62 Å, respectively, demonstrating good agreement with experimental values (FePt: 3.83 Å; Cu: 3.59 Å).^35,36^ Additionally, the Climbing Image Nudged Elastic Band (CI-NEB) method^37,38^ was utilized to identify transition states and minimum energy paths for all reactions. The species′ adsorption energy (*E*_ads_) on surfaces was determined using the formula:
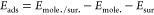
where *E*_sur._ represents the total energy of the metal and core–shell systems, *E*_mole._ denotes the total energy of the gas-phase molecule, and *E*_mole./sur._ corresponds to the total energy of the metal and core–shell systems in the presence of the adsorbate.

### 2.12 SA-Modified FePt@Cu/Au Nanocubes

A solution of FePt@Cu/Au nanocubes (1000 ppm in Cu) in 0.5 mL of ethanol was combined with 0.5% stearic acid (SA) and subjected to sonication for 10 min. Following this, 1 mL of water was introduced to the solution, which was then sonicated for an additional 10 min. The mixture was subsequently centrifuged at 8000 rpm for 5 min and rinsed with water to eliminate any surplus SA. The resulting sediment was resuspended and adjusted in concentration as necessary for the intended experiments.

### 2.13 Evaluation of H_2_O_2_ Generation

To detect H_2_O_2_, a quantitative analysis of hydrogen peroxide in nanocubes was conducted using a hydrogen peroxide assay kit. A calibration curve was established using serial dilutions of H_2_O_2_ at a concentration of 300 μM. The nanocubes were incubated with the hydrogen peroxide assay solution for 10 min. Subsequently, their fluorescence was recorded at an emission wavelength of 510 nm (with an excitation wavelength of 490 nm) using a spectrofluorometer. For comparison, a control measurement was also performed with the hydrogen peroxide assay kit solution in PBS without nanocubes.

### 2.14 Evaluation of ^•^OH Generation Capability

The generation of ^•^OH by FePt@Cu/Au nanocubes in phosphate-buffered saline (PBS) with varying pH levels was assessed by using terephthalic acid (TPA) as a probe. Nanocubes were prepared in PBS with pH values of 5 and 7 and then combined with a 0.1 M solution of TPA for 10 min. The concentrations for Cu, Cu + FePt, FePt@Cu, and FePt@Cu/Au were consistently set at 20 ppm of Cu. For the FePt, Cu+FePt, and FePt + H_2_O_2_ groups, the Fe concentration was fixed at 0.2 ppm. The concentration ratio of Cu and Fe in FePt@Cu of 100:1 was calculated by ICP-AES measurements. Following this incubation, the fluorescence of the TPA was recorded at an emission wavelength of 425 nm (and an excitation wavelength of 315 nm) by a spectrofluorometer. Additionally, a control experiment was conducted using only the TPA solution in PBS, without any nanocubes.

### 2.15 Monitoring the Degradation of FePt@Cu/Au Nanocubes in Acidic Environment

The degradation of FePt@Cu/Au and FePt@Cu/Au@SA nanocubes in acidic environments was tracked over time by using transmission electron microscopy (TEM). The nanocubes were dispersed in phosphate-buffered saline (PBS) at pH 7 and 5, as well as in deionized water, within Eppendorf tubes. These samples were then incubated at 37 °C and monitored over a period of 1 day.

### 2.16 *In Vitro* Cytotoxicity Test

The cytotoxicity of FePt@Cu/Au@SA nanocubes toward the HepG2-Red-FLuc hepatocellular carcinoma cell line was evaluated using the standard methyl thiazolyltetrazolium (MTT) assay. Cells were seeded in 96-well plates at a density of 1 × 10^5^ cells per well and cultured for 24 h in complete media. Subsequently, the cells were treated with varying concentrations of copper in the nanocubes at 37 °C in a 5% CO_2_ atmosphere for 24 h. Following treatment, cells were washed with PBS buffer, and fresh media containing MTT reagent (0.5 mg/mL) was added, followed by incubation for an additional 4 h. The medium was then replaced with DMSO to solubilize the formed formazan. The absorbance of the solution was measured at 540 nm using an ELISA reader.

### 2.17 Live and Dead Cells Assay

Propidium iodide (PI) and Calcein-AM dyes were utilized to distinguish between dead and living cells, respectively. HepG2-Red-FLuc cancer cells were plated in 96-well plates at a density of 8000 cells per well and incubated for 24 h. Subsequently, the cells were treated either with the medium alone (as a blank) or with 100 ppm of FePt@Cu/Au@SA nanocubes for an additional 24 h. Following the treatment, the cells were washed gently twice before being stained with PI and Calcein-AM according to the established protocol. The distribution of dead and living cells was then examined by using a laser scanning confocal microscope.

### 2.18 Flow Cytometry Assay

HepG2-Red-FLuc hepatocellular carcinoma cells were cultured in a 6 cm dish, starting with a density of 5 × 10^5^ cells, and allowed to incubate overnight. The cells were subsequently exposed to 100 ppm of FePt@Cu/Au@SA nanocubes. For comparison, control groups were set up: one with just the culture medium as a negative control and another with 2 μM thapsigargin serving as a positive control. After a 24 h period, the cells were washed twice with PBS and detached using trypsinization. Following detachment, the cells were collected and given a PBS wash. They were then resuspended in 500 μL of 1× annexin-V binding buffer. To this suspension, 10 μL of annexin-V (FITC) and 10 μL of propidium iodide were added. The cells were incubated at room temperature for 15 min before being subjected to flow cytometry analysis. Initial gating of cell populations was performed using a forward scatter and side scatter plot from a cell-only sample to exclude dead cells and cell aggregates. This gating strategy was then consistently applied across all samples for analysis.

### 2.19 *In Vitro* Cu^+^ Detection

HepG2-Red-FLuc cancer cells were cultured in 8-well plates at a density of 10 000 cells per well and allowed to incubate for 24 h. Subsequently, the cells were treated with 5 μM CopperGreen dyes in conjunction with FePt@Cu/Au@SA nanocubes for an additional period of 24 h. A control group was treated with only the culture medium. Following these treatments, the cells were carefully washed twice in preparation for examination using a laser scanning confocal microscope.

### 2.20 *In Vitro* H_2_O_2_ Detection

HepG2-Red-FLuc cancer cells were plated in 8-well plates, with each well containing 10 000 cells, and incubated for 24 h. After this incubation period, the cells were subjected to treatment with a Hydrogen Peroxide Assay Kit (5 μM) along with FePt@Cu/Au@SA nanocubes for an additional duration of 24 h. For the control group, cells were treated solely with the culture medium. Following treatment, the cells were carefully washed twice to prepare them for subsequent analysis using a laser scanning confocal microscope.

### 2.21 *In Vitro*^•^OH Detection

HepG2-Red-FLuc cancer cells were cultured in 8-well plates at a concentration of 10 000 cells per well and incubated for 24 h. Subsequently, they were exposed to treatments combining 5 μM APF dyes with FePt@Cu/Au@SA nanocubes for a period of 24 h. A control group received only the culture medium treatment. After these treatments, the cells were delicately washed twice, setting the stage for additional examination via laser scanning confocal microscopy.

### 2.22 Hemolysis Analysis

Red blood cells at a concentration of 2% were suspended in deionized water (serving as the positive control group), PBS (acting as the negative control group), and PBS mixed with 20 ppm of FePt@Cu/Au@SA nanocubes. These mixtures were kept in the dark for 1 h. Subsequently, they were subjected to centrifugation at 8000 rpm for 5 min to assess the degree of hemolysis.

### 2.23 Biosafety Study

Male C57BL/6 mice aged 6–8 weeks received either 100 μL of sterile PBS or 100 μL of 600 ppm FePt@Cu/Au@SA (dissolved in sterile PBS) through intravenous administration. Following the treatment, the daily body weight of each group was recorded. Additionally, on day seven post-treatment, experimental mice were sacrificed, and samples of blood and normal organs (i.e., heart, lung, spleen, liver, and kidney) were collected for serum biochemical analysis and H&E staining.

### 2.24 Hematoxylin and Eosin (H&E) Staining

The tumor and normal organ samples, including the heart, lung, spleen, liver, and kidney, were embedded in paraffin and sliced into 5 μm thickness. The sections underwent deparaffinization, rehydration, PBS washing, and staining with hematoxylin solution (Merck) for 3 min. After rinsing in tap water, an eosin solution (Merck) was applied for 1 min. Subsequently, the sections were immersed in ethanol and xylene before being mounted for evaluation. The sections were examined under a BX51 microscope (Olympus), which captured images from three different fields for each group.

### 2.25 Serum Biochemical Analysis

The mice’s blood was collected from the heart, and heparin sodium was promptly added. The gathered blood samples underwent centrifugation at 3000 rpm for 10 min to acquire the serum. The obtained serum samples were employed for blood biochemistry analysis, measuring the expression of alkaline phosphatase (ALP), alanine aminotransferase (ALT), aspartate aminotransferase (AST), blood urea nitrogen (BUN), creatine (CREA), total bilirubin (T-Bil), and uric acid (UA) using a FUJI DRI-CHEM 4000i (FUJIFILM).

### 2.26 Biodistribution *In Vivo*

Female SCID mice, aged between 4 and 7 weeks, were sourced from the Laboratory Animal Center at National Cheng Kung University, Taiwan. A solution containing FePt@Cu/Au@SA nanocubes (with a Cu concentration of 600 ppm and volume of 100 μL) was intravenously administered to the SCID mice, with three mice per experimental group. A control group receiving only PBS was also included in the study. Subsequent to the treatment, major organs of the mice, including the heart, liver, spleen, lungs, and kidneys, along with urine samples, were collected, weighed, and then analyzed for Cu content using inductively coupled plasma atomic emission spectroscopy (ICP-AES).

### 2.27 Establishment of Orthotopic Hepatocellular Carcinoma Mice

Male NOD-SCID mice, aged 6–8 weeks, were anesthetized with an intraperitoneal injection of Zoletil 100 (Virbac) and placed supine. Subsequently, 2 × 10^6^ HepG2-Red-Fluc cells, suspended in a solution comprising 10 μL of PBS and 10 μL of Basement Membrane Matrix (BD Biosciences), were surgically implanted into either the right or left lobe of the liver using BD Insulin Syringes 30G 3/10 cm^3^ (BD Biosciences). The incision was closed with CT204 Chromic Catgut (20 mm, 75 cm, UNIK SURGICAL SUTURES MFG. CO.) and NC193 Monofilament Nylon (19 mm, 45 cm, UNIK SURGICAL SUTURES MFG. CO.). The mice were then allowed time to rest until they fully recovered. The IACUC of NCKU set a maximum allowable tumor burden, specifying that the tumor’s weight should not exceed 10% of the body weight, and ascites formation should not be present. All experimental mice with orthotopic hepatocellular carcinoma were euthanized before reaching the criteria mentioned above.

### 2.28 Antitumor Efficacy Study

Orthotopic HepG2-Red-FLuc hepatocellular carcinoma mice were treated with a single dose of either 100 μL of sterile PBS or 100 μL of the following 600 ppm of NPs, including FePt@SA, Cu@SA, FePt@Cu@SA, and FePt@Cu/Au@SA (dissolved in sterile PBS) through intravenous administration. After the treatment, the body weight of each group was documented, and the progression of tumor growth in HepG2-Red-FLuc hepatocellular carcinoma cells was tracked using the IVIS system twice a week. On day 25 post-treatment, experimental mice were sacrificed, and the liver samples with HepG2-Red-FLuc hepatocellular carcinoma were harvested for H&E staining and IHC staining.

### 2.29 IVIS System and Quantification

The mice were anesthetized using oxygen and isoflurane, followed by intraperitoneal injection of 100 μL of D-luciferin (Caliper Life Sciences). After 10 min, the mice underwent imaging with the Xenogen IVISR Spectrum Noninvasive Quantitative Molecular Imaging System (IVIS) (Caliper Life Sciences) at an emission wavelength of 560 nm. The obtained images were analyzed by using Living Imaging software (Caliper Life Sciences).

### 2.30 Immunohistochemistry (IHC) Staining

The tumor samples were embedded in paraffin and sliced into 5 μm thickness. The sections underwent deparaffinization and rehydration and were then incubated with phospho-histone H2A.X (Ser139) antibody (Cell Signaling Technology) or cleaved caspase-3 (Asp175) antibody (Cell Signaling Technology). Subsequently, staining was performed using an ABC peroxidase standard staining kit (Thermo Fisher Scientific) containing biotinylated affinity-purified goat antirabbit IgG (Thermo Fisher Scientific) and a DAB peroxidase (HRP) substrate kit (Vector Laboratories), following the manufacturer’s protocol. Finally, the sections were examined under a BX51 microscope (Olympus), with three different fields captured for each group.

### 2.31 Statistical Analysis

All experiments were independently carried out in triplicate, and the results were presented as the mean ± standard deviation (SD). Differences between groups were compared using one-way ANOVA, and a *p* value below 0.05 indicated a statistically significant difference.

## 3 Results and Discussion

### 3.1 Characterization of FePt@Cu and FePt@Cu/Au Nanocubes

**Figure 1:**
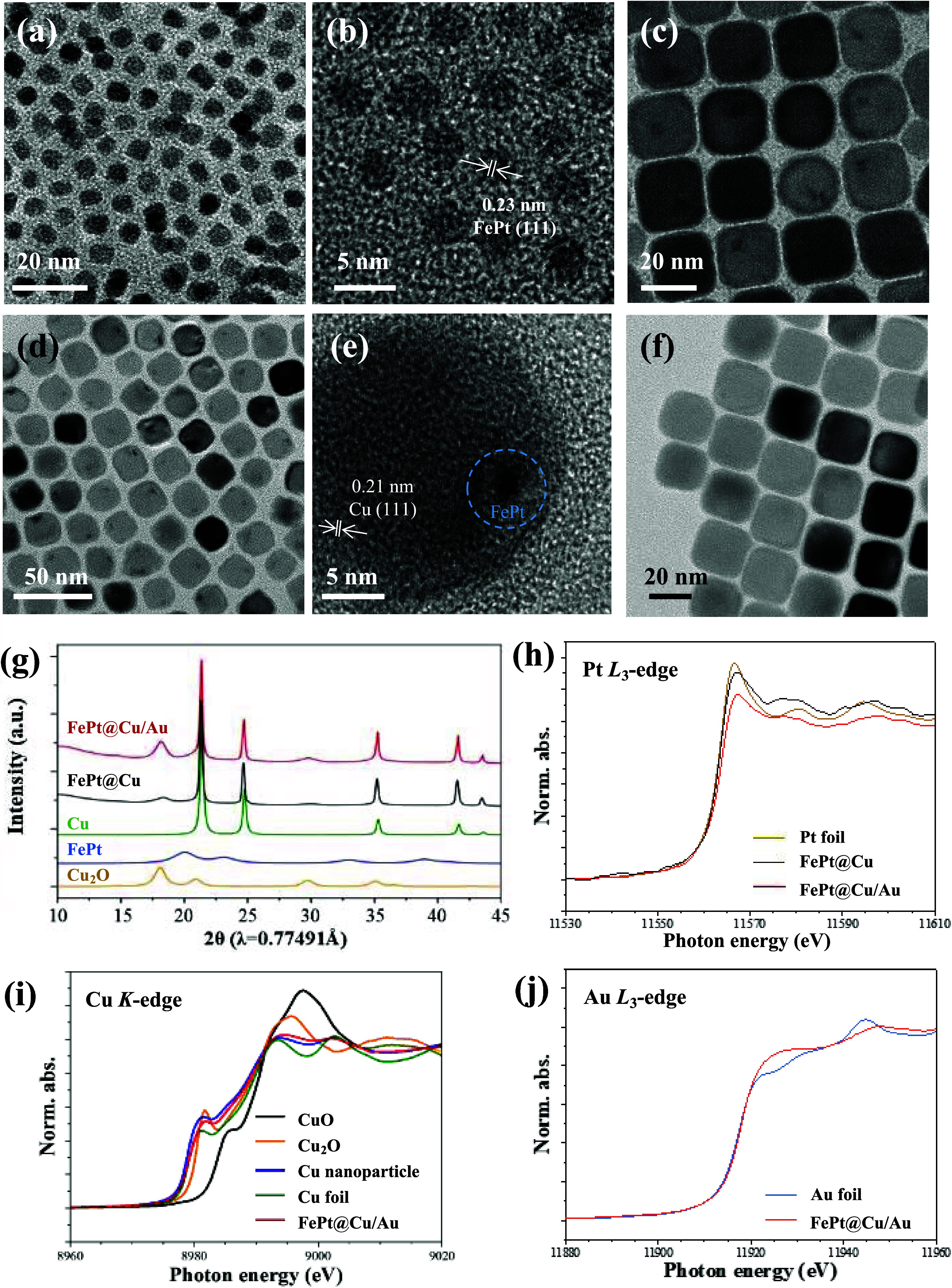
Characterization of nanoparticles. (a) TEM images of FePt nanocubes. (b) HR-TEM image of FePt nanocubes. (c) TEM image of FePt@Cu nanocubes. (d) TEM images of FePt@Cu/Au nanocubes. (e) HRTEM image of FePt@Cu/Au, the lattice space correlates to the Cu(111) phase and the blue circle indicates FePt nanocube. (f) TEM image of compared Cu nanocubes. (g) XRD results of FePt, Cu, Cu_2_O, FePt@Cu, and FePt@Cu/Au. (h) Pt L_3__edge XANES spectra of FePt@Cu/Au, FePt@Cu and Pt foil. (i) Cu K_edge XANES spectra of FePt@Cu/Au, Cu nanoparticles, Cu foil, and cuprous oxides. (j) Au L_3__edge XANES spectra of FePt@ Cu/Au and Au foil.

The fabrication of FePt nanoparticles involved a precisely controlled thermal decomposition process, wherein platinum acetylacetonate and Fe(CO)_5_ precursors were heated to a maintained temperature of 220 °C.^39^ The nanoparticles with uniform morphology and composition are shown in Figures 1a and S1. The FePt nanoparticles exhibit an edge length of approximately 5 nm (based on TEM measurements), with the Fe to Pt ratio determined as 50.1:49.9, as evidenced by EDS results. In addition, the high-resolution TEM (HR-TEM) images displayed a crystalline structure with a lattice spacing of 0.23 nm, corresponding to the (111) face of FePt (Figures 1b and S1a). Subsequently, the as-synthesized FePt nanoparticles acted as nucleation sites for the heterogeneous growth of the Cu nanocube, leading to the formation of the core–shell structured FePt@Cu nanocubes. As seen in Figure 1c, the TEM images show the well-defined core–shell structure of FePt@Cu nanocubes with an edge length of 21 ± 0.2 nm, depicting a cubic morphology and a distinct dark contrast of FePt in the Cu nanocubes. Following the oil/water phase transformation procedure, the mixture of cetyltrimethylammonium bromide (CTAB) and polyvinylpyrrolidone (PVP) was used to transfer the oil-phase FePt@Cu nanocubes into the water phase. Single Au atoms anchored FePt@Cu nanocubes were executed through a galvanic replacement reaction by adjusting the amount of precursor HAuCl_4_^–^.^18^ Thereafter, the oxidized Cu atoms were replaced by Au atoms owing to the different redox potential between Cu^2+^/Cu (0.34 V vs the standard hydrogen electrode [SHE]) and AuCl_4_^–^/Au (0.99 V vs SHE). The FePt@Cu/Au nanocubes maintained a cubic morphology with the edge length remaining at 20 nm (Figure 1d) and displayed crystalline structure with the (111) face corresponding to Cu lattice spacing of 0.21 nm (Figure 1e). The EDS mapping and TEM image of a single FePt@Cu/Au are shown in Figure S2a. The results display an obvious Cu signal but less Fe, Pt, and Au signals. Cu atoms present a relatively higher percentage of an element in FePt@Cu/Au than the amounts of Pt, Fe, and Au. For further evidence, we utilized the EDS-point method to measure the elemental composition in FePt@Cu/Au nanocube (Figure S2b). The results show an element ratio of Cu, Fe, Pt, and Au of 97.5, 1.2, 1.1, and 0.2, respectively. These findings support the Fe, Pt, Cu, and Au species in the FePt@Cu/Au. For comparison, pure Cu and Au nanocubes were also prepared as control groups for further studies (Figures 1f and S3). The Au, Cu nanocubes, FePt@Cu nanocubes, and FePt@Cu/Au nanocubes exhibited the surface plasmon resonance (SPR) bands at 535, 580, 580, and 600 nm, respectively (Figure S4).

**Figure 2:**
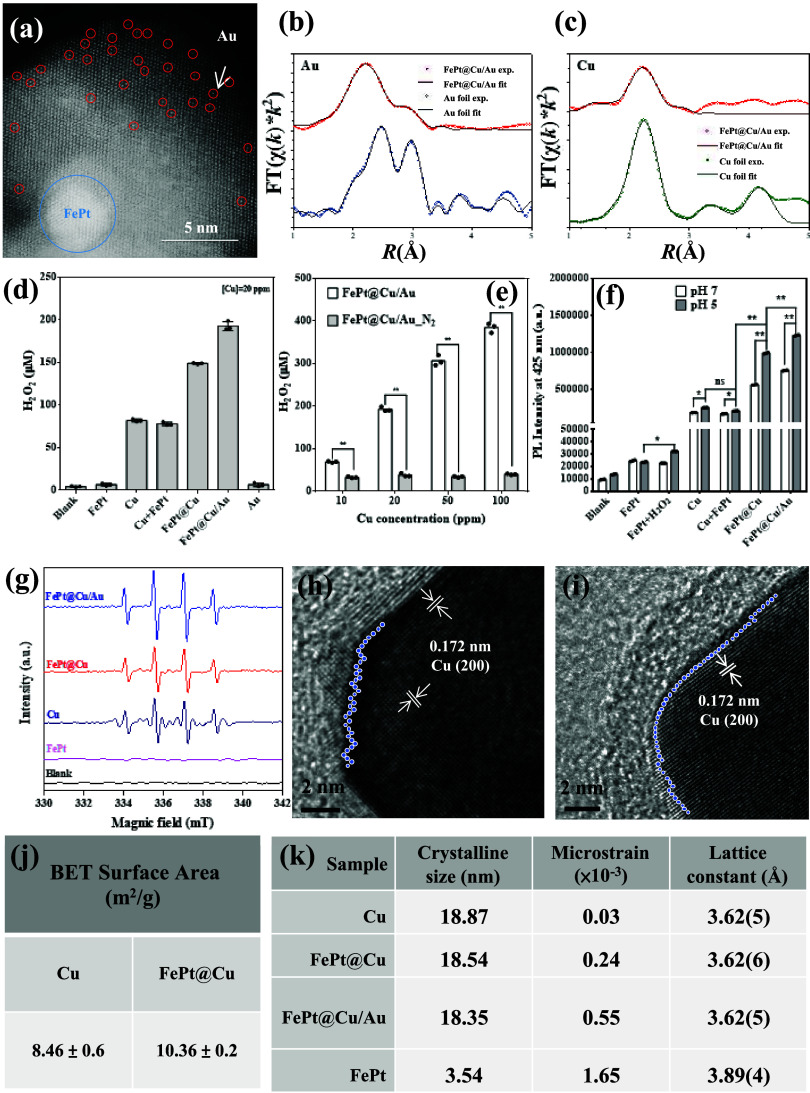
Synergetic self-supply of H_2_O_2_ and ^•^OH generation from FePt@Au/Cu. (a) HAADF-STEM image showing the single-atom property of FePt@Cu/Au nanocube. (b) FT-EXAFS spectra of Au L_3__edge for FePt@Cu/Au and Au foil. (c) FT-EXAFS spectra of Cu K_edge for FePt@Cu/Au and Cu foils. All data were obtained in triplicate. (d) Quantification of H_2_O_2_ generation using H_2_O_2_ kits. (e) H_2_O_2_ generation efficiency at different concentrations under normal and anaerobic conditions. (f) ^•^OH efficiency at different pH levels (5 and 7) detected by TPA fluorescence intensity. (g) The 1:2:2:1 amplitude with quartet ESR signals of DMPO–OH associated with ^•^OH from FePt, Cu, FePt@Cu, and FePt@Cu/Au nanocubes. (h) HRTEM image of the edge of FePt@Cu nanocube; blue dots represent surface alignment, indicating a rough surface. (i) HRTEM image of the edge of Cu nanocube; blue dots represent surface alignment, indicating a smooth surface. (j) BET results showing the surface area of core–shell FePt@Cu and Cu nanocubes. (k) Voigt fit by XRD; the slope indicates the strain of the crystal (the *p*-values calculated by one-way ANOVA: *p < 0.05, **p < 0.01, ns: no significance).

Synchrotron powder X-ray diffraction (XRD) results offer valuable insights into the crystal structure. Figure 1g illustrates that the primary phase of Cu in these nanocubes aligns with the face-centered cubic (FCC) crystal structure, consistent with the information documented in the copper crystal file (ICSD 47614). Additionally, the FePt@Cu and FePt@Cu/Au structures display trace amounts of cuprous oxide, as observed in ICSD 47612. TEM images confirm the growth of gold atoms on the surface of FePt@Cu nanocubes (Figure 2a). Moreover, due to the higher concentration of Cu atoms in the Au/Cu shell, the FePt nanoparticles, acting as the core, are barely discernible in the PXRD pattern of samples FePt@Cu and FePt@Cu/Au, as corroborated by TEM images. In order to further study amorphous materials to reveal the chemical environment and coordination of Au atoms, we applied the X-ray absorption spectroscopy (XAS) technique. XAS includes two main techniques: X-ray absorption near-edge spectroscopy (XANES) and extended X-ray absorption fine structure (EXAFS). XANES reveals information about the local electronic and structural properties of materials near the absorption edge of a specific element. As shown in Figure 1h, Pt absorption edges of the sample of FePt@Cu and FePt@Cu/Au almost overlaid with Pt metal foils indicate that Pt in the nanocube is in zero valence inset of a serial processing in synthesis core–shell structure. The absorption edge of Cu nanoparticle in Cu K-edge XANES is close to Cu foil, indicating the copper atom existed zero valence, while FePt@Cu/Au is located between Cu foil and Cu_2_O indicating that Cu has slightly oxidized (Figure 1i). The Au XANES spectra in FePt@Cu/Au present an interesting result in which the absorption edge overlapped with Au foil while the white lines at 11924 eV show higher intensity (Figure 1j). This change can be viewed as a modification of the Au 5d occupied/unoccupied (d hole) state, representing the charge transfer between Au and Cu. The higher intensity indicates an increased number of vacancies in the Au 5d band due to interactions with Cu.^40^

### 3.2 Characterization of Single-Atom Au Dispersed on Cu

To further characterize FePt@Cu/Au, aberration-corrected high-angle annular dark-field scanning transmission electron microscopy (AC-HAADF-STEM) is used to observe the atomic level, showing single Au atoms (marked by red circles) on the FePt@Cu nanocubes’ surface (Figure 2a) with the magnified view of Figure 2a in Figure S5. Although atomic-resolution STEM offers direct single-atom composition visualization, it is limited to specific local areas. Conversely, XAS is element-sensitive and assesses microcrystalline and amorphous materials in a broader sample range. EXAFS provides detailed insights into the local atomic environment beyond the absorption edge. The EXAFS spectra of the Au L_3_-edge and Cu K-edge of FePt@Cu/Au nanocubes are displayed in Figure 2b,c. We employed Fourier transform (FT) to convert from k-space to R-space, enabling the analysis of the coordination number (CN) and atomic distance (R) of Cu and Au atoms in FePt@Cu/Au from their EXAFS data. As illustrated in Figure S6, the first shell of Cu–Cu with a coordination number of about 5.2 is located at 2.54 Å, which is shorter than that of Cu metal foil (2.59 Å), indicating compression of Cu atoms by electron-rich Au atoms. The low coordination number (3.2) in Cu K-edge EXAFS is attributed to the strong signal from Cu nanocrystallites overshadowing the weak Cu–Au signal. Conversely, the coordination number of Au–Cu from the Au L_3_-edge is 6.4, indicating a highly dispersed distribution of Au atoms on the copper crystal lattice. The result suggests facile galvanic replacement between Au and FePt@Cu nanocubes, leading to the formation of FePt@ Cu/Au nanocubes. Furthermore, the distance of Au–Cu (2.62–2.79 Å) is longer than Cu–Cu (2.59 Å) yet shorter than Au–Au (2.86 Å), potentially inducing tensile strain in Cu atoms coordinated to Au, which can possibly enhance chemical reactivity.^25^ Detailed coordination numbers, path distances, and fitting details are presented in Figure S7 and Tables S1 and S2.^41^

### 3.3 Self-Supplying H_2_O_2_ from Aerobic O_2_ and Fenton-like Reaction

The X-ray absorption spectrum is the sum of the contributions of all of the target elements in the sample. Cu XANES (Figure 1i) in FePt@Cu/Au show slight oxidation, which is mainly contributed by zerovalent Cu and trace amounts of 1+ from Cu_2_O. This result is consistent with the XRD in Figure 1g, which also shows major Cu nanocrystals and trace amounts of Cu_2_O crystals. The Cu XANES suggests that Cu in FePt@Cu/Au is mainly in zerovalent. The zerovalent Cu thermodynamically enables to reduce O_2_ to H_2_O_2_ since zerovalent Cu nanocubes′ reduction potentials (+0.522 eV, Cu^+^/Cu; +0.341 eV, Cu^2+^/Cu) are more negative than that of O_2_/H_2_O_2_ (+0.695 eV). The intensity analysis of the H_2_O_2_ response is depicted in Figure 2d. Utilizing a H_2_O_2_ assay kit, H_2_O_2_ generation was quantified across various nanocube groups (FePt, Cu, Cu+FePt, FePt@Cu, FePt@Cu/Au, and Au). The highest H_2_O_2_ generation was observed at 200 μM for a 20 ppm of Cu concentration in FePt@Cu/Au after a 10 min reaction period. The amount of H_2_O_2_ generated in FePt@Cu/Au surpasses the levels of endogenous H_2_O_2_ in tumoral microenvironments (∼100 μM) and is produced in the presence of O_2_. Quantitative results for FePt@Cu/Au demonstrate a 2.4-fold increase in H_2_O_2_ generation compared to both Cu and Cu + FePt, and a 1.28-fold increase compared to FePt@Cu. However, there is no H_2_O_2_ production in the Au and FePt nanoparticles. To confirm the *in situ* generation of H_2_O_2_ from O_2_ in an aerobic environment, the experiments included incubating FePt@Cu/Au nanocubes with an H_2_O_2_ assay kit under N_2_-filled (anaerobic) conditions and ambient conditions (aerobic). Figure 2e illustrates the enhanced H_2_O_2_ generation at varying concentrations of FePt@Cu/Au nanocubes under ambient conditions, as opposed to N_2_-filled conditions. Cu acts as a potential candidate for triggering H_2_O_2_ generation and formation of ^•^OH radicals through Fenton-like reactions. Figure 2f illustrates the generation of ^•^OH radicals, as indicated by the increased fluorescence emission (λ_em_ = 425 nm) observed using the terephthalic acid (TPA) probe at varying pH levels (pH = 5 and 7). Comparison ^•^OH production of various nanocube groups: FePt@Cu/Au produced more ^•^OH than other nanocube groups. Hence, FePt@Cu/Au is a promising chemodynamic agent for O_2_ → H_2_O_2_ → ^•^OH reactions. The ^•^OH generation capacity of the Cu group is comparable to that of the physical FePt+Cu mixture. While FePt nanoparticles mixed with H_2_O_2_ can convert H_2_O_2_ to ^•^OH, their efficiency is low. Overall, both core–shelled structures in the FePt@Cu and FePt@Cu/Au groups exhibit superior ^•^OH and H_2_O_2_ generation. These core–shell constructs demonstrate a synergistic effect in ^•^OH production, where the result surpasses the sum of individual contributions (1 + 1 > 2). That is the core–shell nanocubes demonstrate a significant enhancement in hydroxyl radical (^•^OH) generation compared to the combined ^•^OH production of individual FePt nanoparticles and Cu nanocubes. Specifically, FePt@Cu nanocubes demonstrate a 3.9-fold increase compared to Cu and a remarkable 42-fold increase over FePt nanoparticles in ^•^OH production under acidic conditions (pH = 5). In the sequential reactions of O_2_ → H_2_O_2_ → ^•^OH, FePt@Au/Cu nanocubes exhibit a 4.8-fold increase in ^•^OH production compared with Cu and a significant 52-fold enhancement over FePt. This systematically explores the synergistic effect in ^•^OH production from combining core–shell and single-atom structures for FePt@Cu/Au. Furthermore, the higher Cu concentration in the FePt@Cu/Au sample exhibits a greater amount of ^•^OH generation under respective pH values of 5 and 7 (Figure S8). We also evaluated the generation of ^•^OH using electron spin resonance (ESR) (Figure 2g). The FePt@Cu/Au exhibits the highest ^•^OH generation intensity compared to those of FePt@Cu, Cu, and FePt. To gain insight into this enhanced reactivity, a more detailed structural analysis was conducted. The HR-TEM image of FePt@Cu indicates that the lattice distance of Cu remains at 0.172 nm, corresponding to the (200) face (Figure 2h,i). However, the edge of the FePt@Cu nanocube exhibits a rough surface and uneven arrangement of Cu atoms together with a lattice mismatch between FePt and Cu of 7%.^42^ This significant mismatch in the core–shell structure induces numerous distortions, leading to the formation of defects when Cu is incorporated. Furthermore, the BET results prove that FePt@Cu nanocubes exhibit a 1.2-fold higher surface area than Cu nanocubes (Figure 2j). To explore the core–shell effect, we employed the Williamson–Hall Plot to calculate the crystal size and lattice microstrain (Figure S7). The pure FePt nanoparticles, with a diameter of approximately 4 nm (based on XRD measurements) and then coated with Cu as the shell, exhibit a larger grain size of around 19 nm. Comparatively, the crystalline size experiences a slight decrease in samples FePt@Cu and FePt@Cu/Au, while the lattice microstrain significantly increases (Figure 2k). Lattice microstrain may stem from microstructural defects, irregularities, or variations in the grain size within the lattice. In the case of the core–shell structure of FePt@Cu/Au, lattice microstrain may slightly affect the arrangement of Cu atoms.^43^ This increased surface area and lattice strain potentially enhance O_2_ absorption and hydrogen peroxide production in core–shell structures, a hypothesis to be theoretically validated later.

### 3.4 Simulation Analysis for Oxidase and Fenton-like Reactions

**Figure 3:**
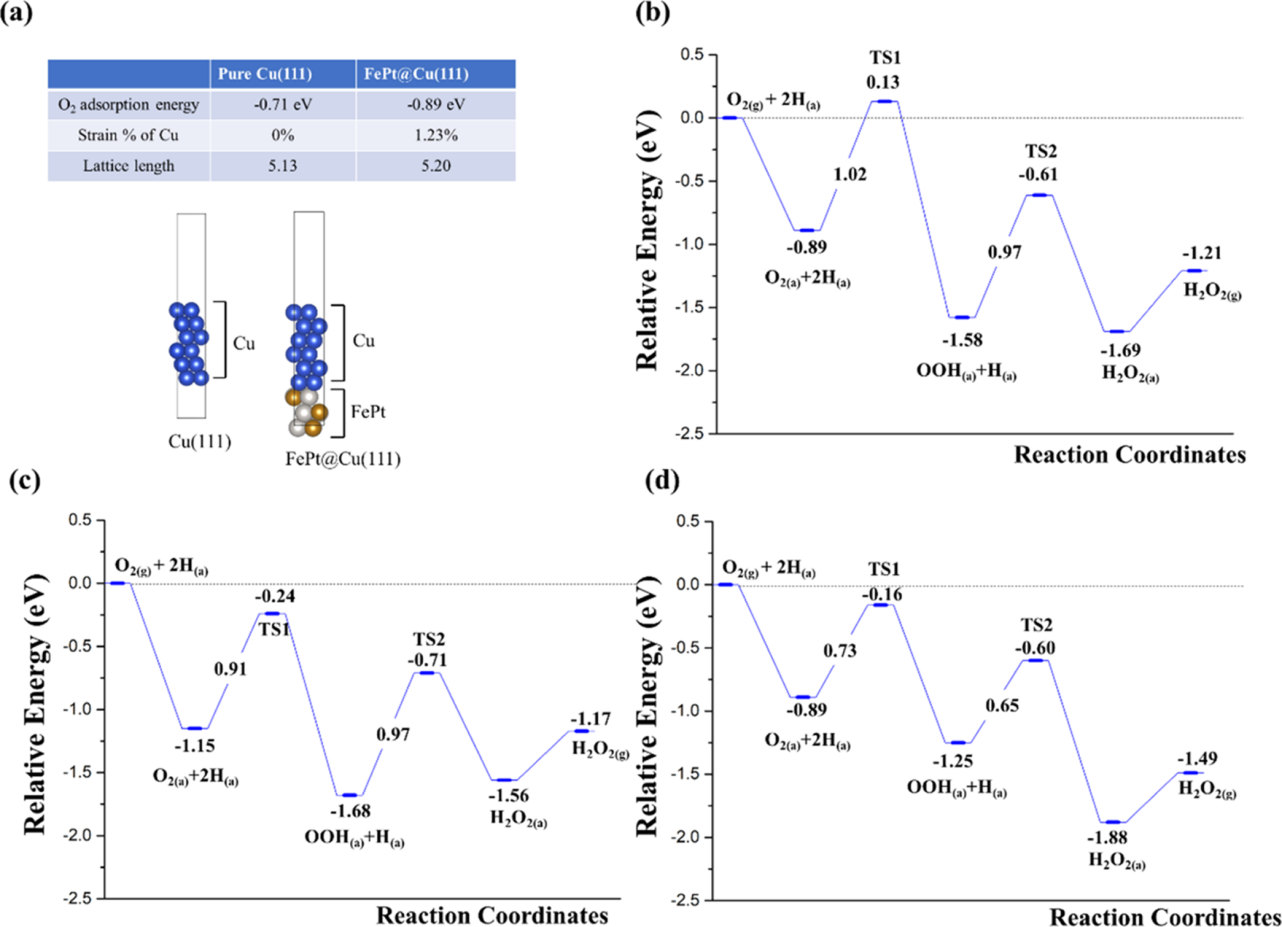
DFT calculations. (a) O_2_ adsorption energy, strain %, lattice length, and model of pure Cu(111) and FePt@Cu(111) surfaces. (b) Calculated potential energy profile of H_2_O_2_ production on pure Cu(111) surface. (c) Calculated potential energy profile of H_2_O_2_ production on FePt@Cu(111) surface. (d) Calculated potential energy profile of H_2_O_2_ production on FePt@Cu/Au(111) surface.

We also calculated the surface reactivity of different metal and core–shell systems using density functional theory (DFT) calculations. Because the lattice constants of bulk Cu and FePt are 3.59 and 3.83 Å, respectively, the lattice strain effects must occur in the FePt@Cu and FePt@Cu/Au core–shell systems. We first calculated and compared the pure metal Cu(111) and core–shell FePt@Cu(111) surfaces concerning the adsorption of O_2_. The optimized lattice length of Cu(111) is 5.13 Å, while the optimized lattice length of FePt@Cu(111) is 5.20 Å. This reveals that FePt@Cu(111) can expand pure Cu(111) by around 1.23%. Besides, we found that the adsorption energy of O_2_ on the Cu(111) is −0.71 eV, while it becomes −0.89 eV on the FePt@Cu(111) surface, as shown in Figure 3a. Because the core size is pretty small and the shell size is large in our experiment, the FePt core structure would not contact the O_2_ molecule during the adsorption. The FePt core makes it difficult to possess electronic interaction with the O_2_ molecule. Therefore, the results show that the strain effects play more important roles in the adsorption of the O_2_ molecules on the FePt@Cu(111) surface. The strain effects can induce the enhancement of the adsorption energy of O_2_ on the FePt@Cu(111) surface compared with the pure Cu(111) surface.

To further understand the reactivity difference between the metal and core–shell systems, we calculated the reaction pathways for the hydrogenation of the O_2_ to H_2_O_2_ on the pure Cu(111), FePt@Cu(111), and FePt@Cu/Au(111) surfaces, as shown in Figure 3b–d, respectively. First, we compare the reactivity between pure Cu(111) and FePt@Cu(111) surfaces, where the coadsorption energies of O_2_ and 2H are −0.89 and −1.15 eV on pure Cu(111) and FePt@Cu(111) surfaces, respectively. Additionally, we observed that the first hydrogenation barrier of O_2_ to OOH is 1.02 eV on pure Cu(111) but decreases to 0.91 eV on the FePt@Cu(111) surface. The second hydrogenation barrier of OOH to H_2_O_2_ is the same on the pure Cu(111) and FePt@Cu(111) surfaces. To elucidate, given that the hydrogenation barrier of O_2_ exceeds the adsorption energy of O_2_ on pure Cu(111), the initial hydrogenation reaction on this surface might face difficulty. Conversely, on the FePt@Cu(111) surface, the hydrogenation barrier of O_2_ is lower than its adsorption energy, facilitating an exothermic process. Thus, the first hydrogenation on the FePt@Cu(111) surface represents a thermodynamically and kinetically favorable reaction. As mentioned above, the strain effect plays an important role in the adsorption of aqueous O_2_ on the FePt@Cu(111) surface. Based on the hydrogenation barrier calculation, it shows that the strain effect can slightly reduce the first hydrogenation barrier of O_2_, facilitating the reactivity enhancement for H_2_O_2_ production. As a result, the DFT results reveal that the strain effect of the core–shell structure can affect the catalytic property of H_2_O_2_ formation.

For the reaction barriers of O_2_ hydrogenation to H_2_O_2_ on the FePt@Cu/Au(111) surface, the calculated first and second barriers are 0.73 and 0.65 eV, respectively. The results indicate that the reactivity of H_2_O_2_ production on the FePt@Cu/Au(111) surface is stronger than that on FePt@Cu(111) as well as pure Cu(111) surfaces. The reason is that the Au atom on the shell of the FePt@Cu/Au(111) surface can lower the energy barrier when the hydrogenation pathway goes through the Au atom, which was proved in our previous studies.^18^ The effect is the so-called synergistic effect of bimetallic SACs. Hence, the DFT results demonstrate that the FePt@Cu/Au(111) surface reveals both the strain effect of the core–shell structure and single-atom effect, leading to the stronger catalytic property for the H_2_O_2_ formation than the FePt@Cu(111) and pure Cu(111) surfaces. The reactivity sequence is FePt@ Cu/Au > FePt@Cu > Cu based on the DFT calculations, providing the same trends as our experimental observations.

We have further compared FePt@Cu/Au with other supported metal catalysts based on the activation energies. In the literature, Pd or PdAu alloys were widely used to synthesize H_2_O_2_ from H_2_ and O_2_. It has been reported that the calculated reaction barriers of O_2_ hydrogenation to OOH on Pd(111) and Au@Pd(111) are 0.92 and 0.85 eV, respectively.^44^ In addition, Yu et al. have calculated that the Pd1/TiO_2_ can catalyze O_2_ hydrogenation to OOH via the activation energy of 0.81 eV.^45^ Our results show that the barrier of O_2_ hydrogenation to OOH as 0.73 eV on FePt@Cu/Au is smaller than the previous studies. On the other hand, although there were some studies reported that the barriers of O_2_ hydrogenation to OOH are small, such as on the PdH(211) and AuPd(211) surfaces, their hydrogenation barriers of OOH to H_2_O_2_ are still larger than 0.70 eV.^46^ For comparison, our calculated activation energy of OOH to H_2_O_2_ is 0.65 eV on the FePt@Cu/Au. These results reveal that the catalytic activity of FePt@Cu/Au is stronger than the catalysts mentioned in the literature due to the smaller kinetic barriers.

### 3.5 The Degradable Nature of the FePt@Cu/Au Nanocubes

**Figure 4:**
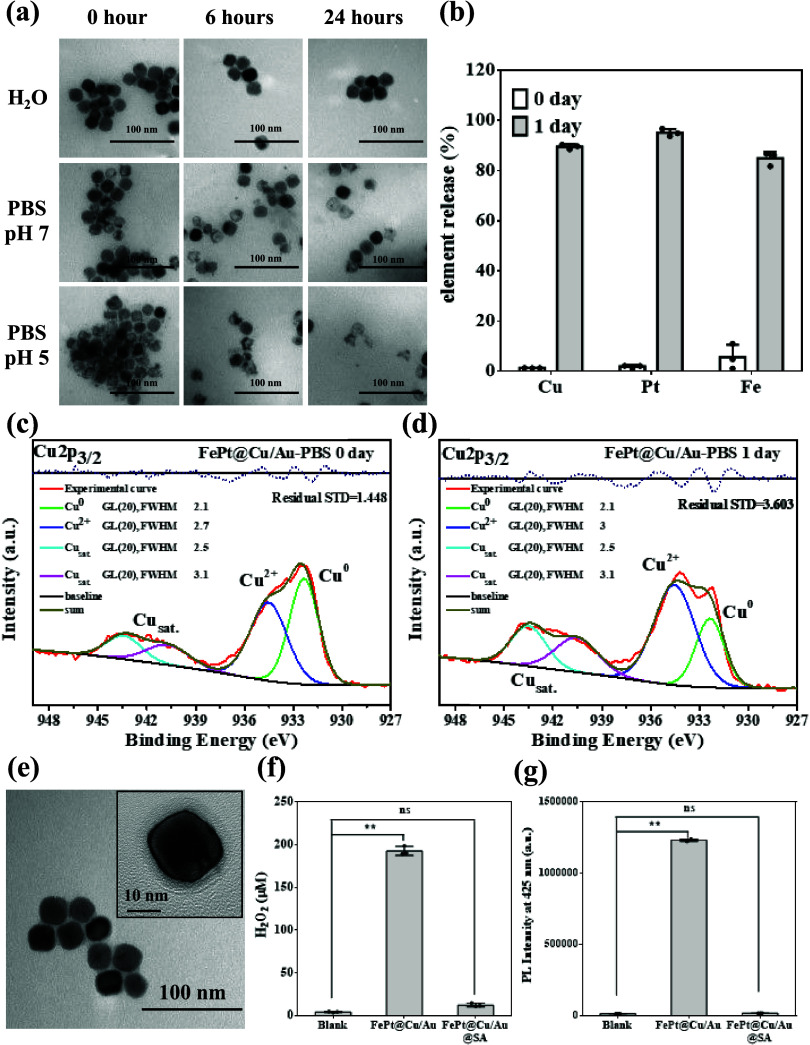
Stability and self-decomposition behavior in FePt@Cu/Au and FePt@ Cu/Au@SA under different conditions (H_2_O, PBS at pH = 7 and 5). (a) TEM images reveal that the FePt@Cu/Au nanocubes started to decompose immediately in PBS. (b) ICP quantized results indicate that over 85% of elements (Cu, Pt, and Fe) were dissolved after 1-day storage in PBS (pH 7). (c, d) The XPS spectrum of FePt@Cu/Au nanocubes given Cu^0^ and Cu^2+^ signals under PBS condition (pH 7) as a function of day (2p_3/2_ assigned as 932 eV for Cu^0^ and assigned as 934 eV for Cu^2+^). (e) TEM image of the FePt@Cu/Au@SA nanocubes. (f, g) Quantitative analysis of H_2_O_2_ and ^•^OH generation in FePt@Cu/Au@SA nanocubes. The suppression of H_2_O_2_ and ^•^OH orignates from the SA modification. All data were obtained in triplicate (the *p*-values calculated by one-way ANOVA: *p < 0.05, **p < 0.01, ns: no significance).

Above the H_2_O_2_ and ^•^OH production results, the chemical reaction shows that Cu^0^ is likely oxidized to Cu^+^ and Cu^2+^. The nanocubes may exhibit dissolution behavior due to the oxidation of Cu. Hence, we monitored the stability of FePt@Cu/Au nanocubes, which show superior H_2_O_2_ and ^•^OH generation, over a period of 1 day in H_2_O, PBS buffer (pH 7), and PBS buffer (pH 5) (Figure 4a). After 6 h incubation, the nanocubes exhibit pronounced decomposition in PBS solution compared to H_2_O, especially in higher-acidity condition of PBS (pH 5). Moreover, the nanocubes accelerate dissolution and nearly completely disintegrated after 1-day in PBS incubation. The quantitative results of Cu, Pt, and Fe ions are shown in Figure 4b, indicating the percentage of element decomposition determined from the supernatant of FePt@Cu/Au nanocubes under PBS (pH 7) over 1 day, measured by ICP. Over 85% of self-decomposing Cu, Pt, and Fe ions were detected, establishing FePt@Cu/Au nanocubes as promising renal-clearable agents for *in vivo* tumor studies. Furthermore, the XPS measurements in Figure 4c,d are used to prove the degradation of FePt@Cu/Au nanocubes after 1 day of incubation in PBS (pH 7). The XPS measurements are determined by the CASA XPS software to fit with the Gaussian–Lorentzian ratio of 20 and demonstrate the residual STD is 1.448 (0-day) and 3.603 (1-day), respectively. Post-1 day, the intensity of Cu^2+^ increased, evidencing the oxidation of Cu^0^ to Cu^2+^. Notably, the self-decomposing behavior could potentially induce oxidative stress during blood circulation *in vivo* due to ^•^OH generation. To mitigate the self-generation of H_2_O_2_ during blood circulation, the surface of the nanocubes was modified with stearic acid (SA). This modification retains the structure of FePt@Cu/Au nanocubes, resulting in dispersed colloidal solutions (Figure 4e). A visible layer of SA coating on the nanocube is evident (inset of Figure 4e). To further confirm the presence of SA on the surface of FePt@Cu/Au nanocubes was demonstrated by FTIR analysis (Figure S9). In the case of surface-modified SA nanocubes, the vibrational peaks at 2916 and 2848 cm^–1^ correspond to the asymmetric and symmetric stretching vibrations of the CH_2_ group, respectively. The peak at 720 cm^–1^ belongs to plane bending of the −CH_2_–. The signal observed at 1560 cm^–1^ represents a downshift from 1704 cm^–1^ (in SA), indicating a change in the stretching frequencies of the C–O bonds within the −COOH groups due to the surface modification of the nanocubes. For the dynamic light scattering (DLS) results (Figure S10a), the hydrodynamic sizes of FePt@CuAu and FePt@CuAu@SA are approximately 24 and 34 nm, respectively, in the H_2_O and the sizes remain the same under different pH values of PBS buffer (pH = 5, 7). FePt@CuAu@SA maintains its DLS hydrodynamic size over a period of 24 h and exhibits excellent stability, retaining its morphological features under different solution conditions (Figure S10a,b). No dissolution of the nanocubes was observed after a 1-day incubation. In Figure 4f,g, we monitored the generation of H_2_O_2_ and ^•^OH to further validate the successful SA (steric acid) coating on the nanocubes using an H_2_O_2_ assay kit and TPA fluorescence probe. Neither the fluorescence intensity from the H_2_O_2_ kit nor the TPA probe was detected in the FePt@Cu/Au@SA treatments, indicating that H_2_O_2_ and ^•^OH generation was successfully suppressed following SA modification. This inhibitory effect can be attributed to the formation of a protective membrane on the FePt@Cu/Au NPs surface, effectively halting the O_2_-driven chemodynamic reactions when particles are circulated in the blood vessel. Notably, when the SA-coated nanoparticles, upon internalization by cancer cells, the SA would fuse with the cell membrane and release the nanoparticles into the cellular environment. Consequently, FePt@Cu/Au NPs are exposed to activate subsequent O_2_-driven chemodynamic reaction reactions within cancer cells.

### 3.6 *In Vitro* Evaluation

**Figure 5:**
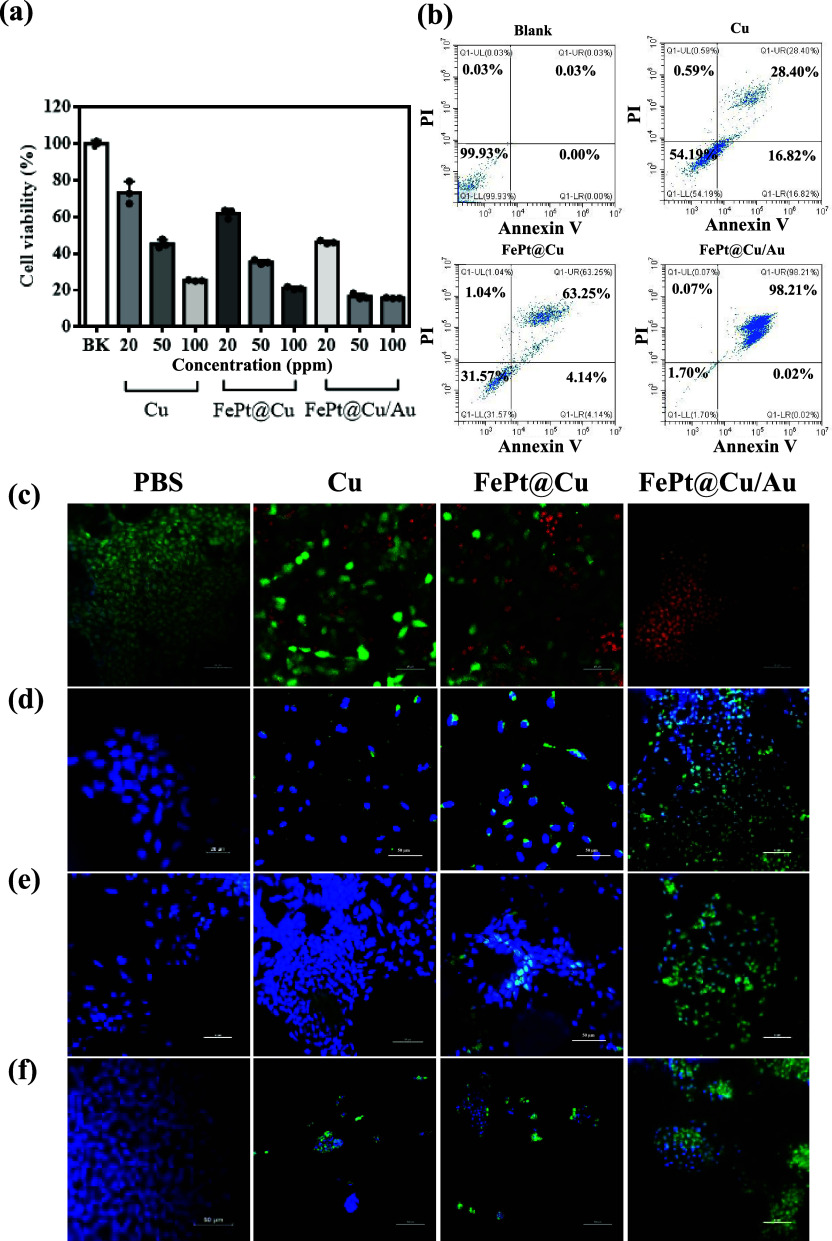
*In vitro* studies of SA coating nanocubes. (a) Cytotoxicity analysis of HepG2 cancer cells treated with Cu@SA, FePt@Cu@SA, and FePt@Cu/Au@SA nanocubes for 24 h incubation. (b) Flow cytometry analysis of HepG2 cancer cells treated with different nanocubes. (c) Live (green color) and dead cells (red color) stained with fluorescent green dye (Calein-AM) and red dye (propidium iodide), respectively, for cancer cells treated with different nanocubes. (d) Cu^+^ release stained by CopperGreen dye treated with different nanocubes showing green color as Cu^+^ releasing. (e) H_2_O_2_ generation stained by hydrogen peroxide assay kit treated with different nanocubes showing green color as H_2_O_2_ generated. (f) ^•^OH generation stained by APF dye treated with different nanocubes showing green color as ^•^OH generated.

The Cu, FePt@Cu, and FePt@Cu/Au were modified with SA for the following *in vitro* studies. An MTT assay evaluates the cell viability of HepG2-Red-FLuc hepatocellular carcinoma cells incubated with Cu@SA, FePt@Cu@SA, and FePt@Cu/Au@SA nanocubes. The MTT assay results, presented in Figure 5a, indicate a concentration-dependent decrease in cell survival rate with increasing concentrations of nanocubes, highlighting FePt@Cu/Au@SA superior efficacy in inducing cytotoxicity compared to that of other groups for cancer cell elimination. Flow cytometry analysis reveals an increase in late apoptosis after 24 h of incubation with FePt@Cu/Au@SA nanocubes (Figure 5b). The cells incubated with FePt@Cu/Au@SA exhibit a relatively higher late apoptotic ratio (98.21%) compared to other groups (cell only: 0.03%, Cu@SA: 28.40%, and FePt@Cu@SA: 63.25%). The morphology of FePt@Cu/Au@SA was disintegrated when cultured with HepG2 cancer cells for 24 h (Figure S11), suggesting the occurrence of the O_2_-driven chemodynamic reactions within cancer cells. Furthermore, the additional HepG2 cells cultured experiments for Cu, FePt@Cu, and FePt@Cu/Au@SA used confocal imaging to observe live and dead cells, Cu^+^ release, H_2_O_2_, and ^•^OH generation. Fluorescence staining experiments on live and dead cells, as illustrated in Figure 5c, provide further evidence of the greater efficacy of FePt@Cu/Au@SA in damaging cancer cells compared with the other groups. The presence of oxidized Cu^+^ ions in cells was verified using CopperGreen dye, emitting green fluorescence (Figure 5d). In Figure 5e,f, FePt@Cu/Au@SA produced higher amounts of H_2_O_2_ and ^•^OH in cells compared to Cu and FePt@Cu. These results indicate that FePt@Cu/Au@SA has a higher efficiency in O_2_-driven chemodynamic therapy *in vitro* compared to the other nanocubes. Importantly, no hemolysis or damage to vascular endothelial cells was observed from FePt@Cu/Au@SA (Figure S12), ensuring its safety during blood circulation.

### 3.7 *In Vivo* Therapeutic Efficacy of FePt@Cu/Au against Hepatocellular Carcinoma

**Figure 6:**
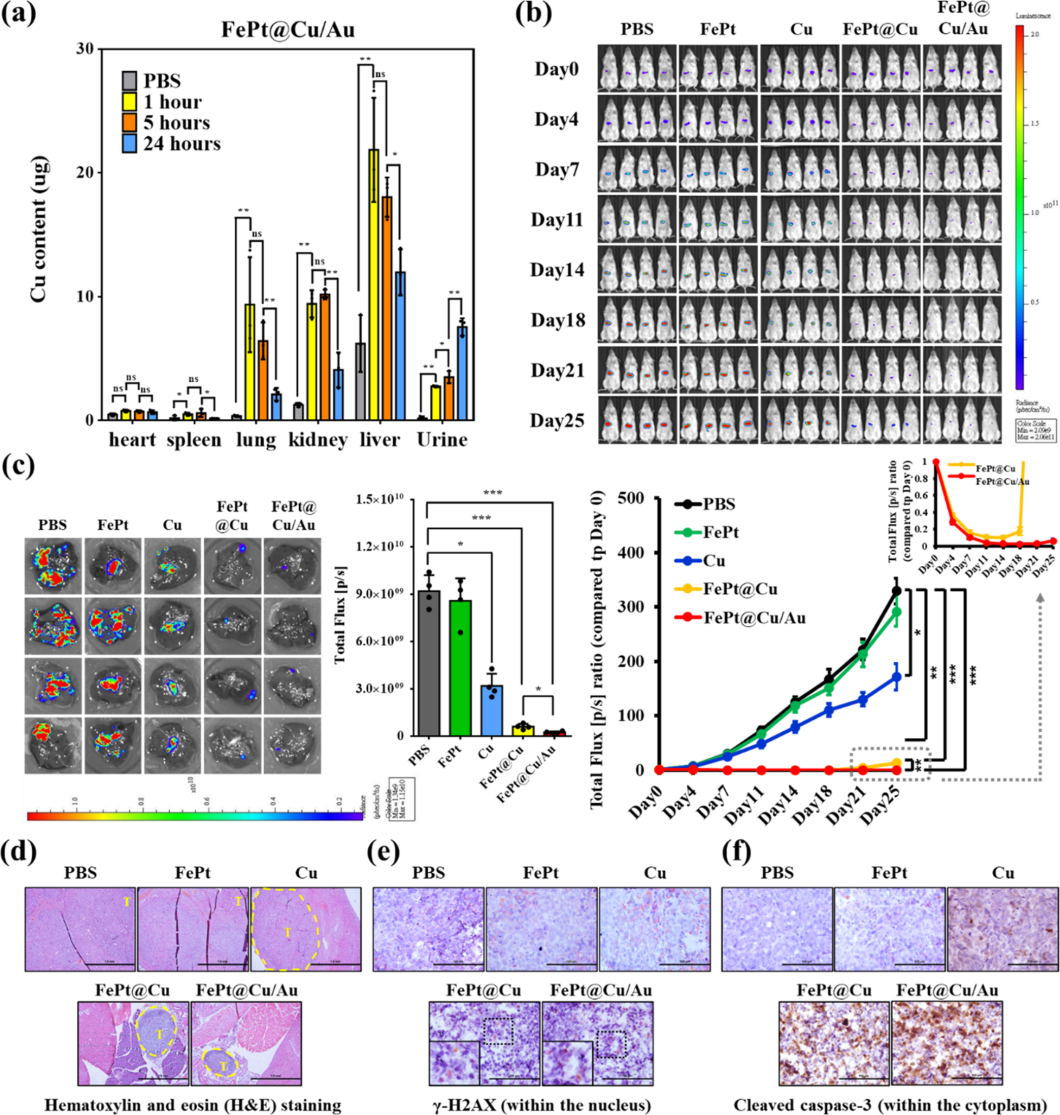
Antitumor efficacy of FePt@Cu/Au@SA in orthotopic hepatocellular carcinoma model (*n* = 4). (a) Biodistribution determined by Cu concentration collected from FePt@Cu/Au@SA nanocubes through intravenous injection. (b) Monitoring the orthotopic tumor growth of HepG2-Red-FLuc cells in NOD-SCID mice treated with PBS, FePt@SA, Cu@SA, FePt@Cu@SA, and FePt@Cu/Au@SA using the IVIS system. (c) Evaluation of IVIS bioluminescence of livers with hepatocellular carcinoma in each treatment group after sacrificing the mice. (d) Assessment of the morphology of hepatocellular carcinoma in each treated mouse through hematoxylin and eosin staining (Scale bar, 1 mm). The tumor area is highlighted by the yellow circle with T labeling. (e) Investigation of the expression of phospho-histone H2A.X (Ser139) and (f) cleaved caspase-3 (Asp175) within hepatocellular carcinoma from mice in each treatment group using IHC staining (Scale bar, 100 μm). The *p*-value was calculated by one-way ANOVA (*p < 0.05, **p < 0.01, ***p < 0.001).

Before conducting the antitumor efficacy experiment, we performed an *in vivo* biosafety study using FePt@Cu/Au@SA in C57BL/6 mice. After 7 days of post-treatment via intravenous (IV) administration, there is no observed impact on murine body weight, serum biochemical indices (i.e., T-Bil, ALP, AST, ALT, BUN, CRE, and UA), and histological features of normal organs, including heart, lungs, liver, spleen, and kidneys, compared to the sterilized PBS control group (Figure S13). This suggests that FePt@Cu/Au@SA exhibits good *in vivo* biosafety, allowing us to proceed with further antitumor experiments. *In vivo* biodistribution indicates the increased Cu element in urine as a function of time, suggesting the decomposition of FePt@Cu/Au@SA to release copper ions given the clearance effect (Figure 6a).

To establish the hepatocellular carcinoma model, we orthotopically injected NOD-SCID mice with HepG2-Red-FLuc cells to induce tumor formation, which was subsequently detected using IVIS systems. The tumor-bearing mice were randomly administered with the following nanocubes coated with SA (one dose), including FePt@SA, Cu@SA, FePt@Cu@SA, and FePt@Cu/Au@SA, via IV injection. They were compared to those of the sterilized PBS control group. As expected, FePt@Cu@SA and FePt@Cu/Au@SA demonstrate significant *in vivo* inhibition of orthotopic HepG2-Red-FLuc tumors compared to the pure FePt and Cu structures (Figure 6b). Tumor regressions were noted in the FePt@Cu@SA group before Day 18 post-treatment. In contrast, the single-atom FePt@Cu/Au@SA group reveals a more pronounced effect, with significant improvements observed before Day 25 post-treatment. Noteworthy, this phenomenon surpassed the performance of our previously reported Au_0.02_Cu_0.98_ nanocubes^18^ underscoring the importance of the core–shell effect. None of the therapeutic groups mentioned above impacted the experimental mice’s body weight (Figure S14).

After the mice were sacrificed on Day 25 post-treatment, *ex vivo* IVIS detection and histological analysis reveal effective tumor inhibition in the FePt@Cu@SA and FePt@Cu/Au@SA groups, as evidenced by the lower luminance signals and the most minor tumor areas indicated by the yellow circle (Figure 6c,d). Once again, the FePt@Cu/Au@SA group exhibits the best antitumor efficacy. According to the above cellular experimental results, the observed phenomenon is attributed to the excessive production of ROS, leading to cancer cell damage. This was evidenced by the abundant expression of γ-H2AX (in the nucleus) and cleaved caspase-3 (in the cytoplasm) in the core–shell FePt@Cu/Au@SA-treated tumor area (Figure 6e,f). These results collectively highlight the exceptional potential of core–shell and single-atom structures in FePt@Cu/Au against hepatocellular carcinoma, providing a new dawn of healing for clinical patients.

## 4 Conclusions

In successfully integrating the catalyst system, we have driven the synergistic effect of core–shell with increased lattice microstrain and single-atom in the FePt@Cu/Au structure. This involves utilizing FePt as a nucleation site for the heterogeneous growth of Cu nanocubes, forming FePt@Cu. Subsequently, a galvanic replacement process is employed to introduce a single-atom Au on the FePt@Cu surface. The presence of atomic Au significantly enhances ^•^OH production, with FePt@Cu/Au demonstrating an impressive 52-fold increase over that of FePt alone. Theoretical analysis reveals a reduced O_2_ adsorption energy and reaction barriers within the core–shell structure, attributable to the lattice mismatch between FePt and Cu and the incorporation of single-atom Au. These factors collectively enhance the O_2_ → H_2_O_2_ → ^•^OH reaction pathway, effectively suppressing tumors. Moreover, the biodegradable nature of the FePt@Cu/Au structure facilitates its excretion through the urinary tract following tail vein administration.

## Supporting Information Available

Curve-fit parameter for Cu K-edge EXAFS for FePt@Cu/Au (Table S1); curve-fit parameter for Au L_3_-edge EXAFS for FePt@Cu/Au (Table S2); HR-TEM image of the FePt nanoparticles and the corresponding EDS signals of Fe and Pt (Figure S1); HR-TEM image of the single FePt@Cu/Au nanocube and the corresponding EDS mapping of Cu, Pt, Au, and Fe (Figure S2); characteristics of Au nanocubes (Figure S3); UV–vis profiles of Au, Cu, FePt@Cu, and FePt@Cu/Au nanocubes (Figure S4); magnified image of Figure 2a showing high resolution of AC-HAADF-STEM image of the FePt@Cu/Au nanocube (Figure S5); k^2^-weighted EXAFS spectra (Figure S6); PXRX pattern fitting and Williamson–Hall plot of samples FePt, Cu, FePt@Cu, and FePt@Cu/Au (Figure S7); efficiency of ^•^OH generation at different pH levels (5 and 7) was detected by measuring TPA fluorescence intensity under varying concentrations (Figure S8); Fourier transform infrared (FTIR) spectra of FePt@Cu/Au nanocubes with stearic acid (SA) modification (Figure S9); DLS size of FePt@Cu/Au and stability of FePt@Cu/Au@SA under different solution conditions, TEM images revealed the stability of FePt@Cu/Au@SA nanocubes under various solution conditions (H_2_O, PBS pH 7, and 5) (Figure S10); morphology of FePt@Cu/Au@SA nanocubes treated with HepG2 cancer cells for 24 h (Figure S11); analysis of hemolysis in blood containing 2% red blood cells from FePt@Cu/Au@SA nanocubes (Figure S12); examining the biosafety of FePt@Cu/Au@SA treatments in C57BL/6 mice (Figure S13); and body weight changes of HepG2-Red-FLuc orthotopic tumor mice in each treatment group (Figure S14) (PDF)
