# Supplementary material for: Synergistic ROS Generation via Core–Shell Nanostructures with Increased Lattice Microstrain Combined with Single-Atom Catalysis for Enhanced Tumor Suppression
Source: ACS Appl Mater Interfaces. 2024 Aug 15;16(34):45356–70. doi: 10.1021/acsami.4c10392 (PMC11367581; doi:10.1021/acsami.4c10392)
Supplement: Supplementary file 1 — am4c10392_si_001.pdf [file am4c10392_si_001.pdf]

Supporting Information

Synergistic ROS Generation via Core-Shell Nanostructures with increased lattice microstrain Combined with Single-Atom Catalysis for Enhanced Tumor Suppression

Liu-Chun Wang[a,b,‡], Li-Chan Chang[c,‡], Hsiang-Lin, Huang[a], Po-Ya Chang[d], Chih-Wen Pao[d], Yin-Fen Liu[c], Keng-Shiang Huang[e], Yi-Hsin Chien[f,], Hwo-Shuenn Sheu[d,*], Wen-Pin Su[b,c,g,h,*], Chen-Hao Yeh[f,*], Chen-Sheng Yeh[a,b,*]*

[a]Department of Chemistry, National Cheng Kung University, Tainan 701, Taiwan

[b]Center of Applied Nanomedicine, National Cheng Kung University, Tainan 701, Taiwan

[c]Institute of Clinical Medicine, College of Medicine, National Cheng Kung University, Tainan 704, Taiwan

[d]National Synchrotron Radiation Research Center, Hsinchu 30076, Taiwan.

[e]The School of Chinese Medicine for Post-Baccalaureate, I-Shou University, Kaohsiung City 82445, Taiwan

[f]Department of Materials Science and Engineering, Feng Chia University, Taichung, 40724, Taiwan

[g]Departments of Oncology and Internal Medicine, National Cheng Kung University Hospital, College of Medicine, National Cheng Kung University, Tainan 704, Taiwan

[h]Clinical Medicine Research Center, National Cheng Kung University Hospital, College of Medicine, National Cheng Kung University, Tainan 704, Taiwan

*Corresponding author e-mail: yhchien@fcu.edu.tw, hsheu@nsrrc.org.tw, wpsu@mail.ncku.edu.tw, chenhyeh@fcu.edu.tw, csyeh@mail.ncku.edu.tw

Table S1: Curve-fit Parameter [a] for Cu K-edge EXAFS for FePt@Cu/Au.

	**Path[b]**	**CN**	**R (Å)**	**σ^2 (Å^2)**
Cu foil	Cu-Cu1	12[c]	2.59(1)	0.009(1)
	Cu-Cu2	6[c]	3.60(2)	0.012(1)
	Cu-Cu3	24[c]	4.46(1)	0.012(1)
	Cu-Cu1-Cu1	48[c]	3.78(3)	0.012(6)
FePt@Cu/Au	Cu-Cu1	5.2(8)	2.54(1)	0.015(6)
	Cu-Au1	3.2(13)	2.79(3)	0.008(1)

[a] The $S0^2$ factor (0.9818) of sample FePt@Cu/Au was applied using a reference Cu foil. ΔE_0 was refined as a global fit parameter, returning a value of 6.5 ± 1.6 eV. The data range covered $3 \leq k \leq 13$ Å^{-1} and $1.6 \leq R \leq 3.2$ Å. There were 7 variable parameter out of a total of 9.9 independent points. The R factor for this fit was 0.6%. [b] The distances for Cu-Cu1 and Cu-Au1 were from the FeFF file of CuAu. [c] The coordination numbers were constrained as follow: N(Cu-Cu1)=12, N(Cu-Cu2)=6, N(Cu-Cu3)=24 and N(Cu-Cu1-Cu1)=48 to determine the relative $S0^2$ applied to the sample FePt@Cu/Au.

Table S2: Curve-fit Parameter [a] for Au L_3-edge EXAFS for FePt@Cu/Au.

	Path[b]	CN	R (Å)	σ^2 ($Å^2$)
Au foil	Au-Au1	12[c]	2.86(0)	0.008(1)
	Au-Au2	6[c]	4.05(3)	0.013(3)
	Au-Au3	24[c]	4.99(3)	0.014(5)
	Au-Au1-Au1	48[c]	4.32(4)	0.006(8)
FePt@Cu/Au	Au-Cu1	6.4(11)	2.62(1)	0.013(3)
	Au-Au1	4.7(11)	2.78(2)	0.011(2)

[a] The $S0^2$ factor (0.7465) of sample FePt@Cu/Au was applied using a reference Au foil. ΔE_0 was refined as a global fit parameter, returning a value of 2.3 ± 1.2 eV. The data range covered $3 \leq k \leq 10$ $Å^{-1}$ and $1.0 \leq R \leq 3.5$ Å. There were 7 variable parameter out of a total of 11.0 independent points. The R factor for this fit was 0.52%. [b] The distances for Au-Cu1 and Au-Au1 were from the FeFF file of CuAu. [c] The coordination numbers were constrained as follow: N(Au-Au1)=12, N(Au-Au2)=6, N(Au-Au3)=24 and N(Au-Au1-Au1)=48 to determine the relative $S0^2$ applied to the sample FePt@Cu/Au.

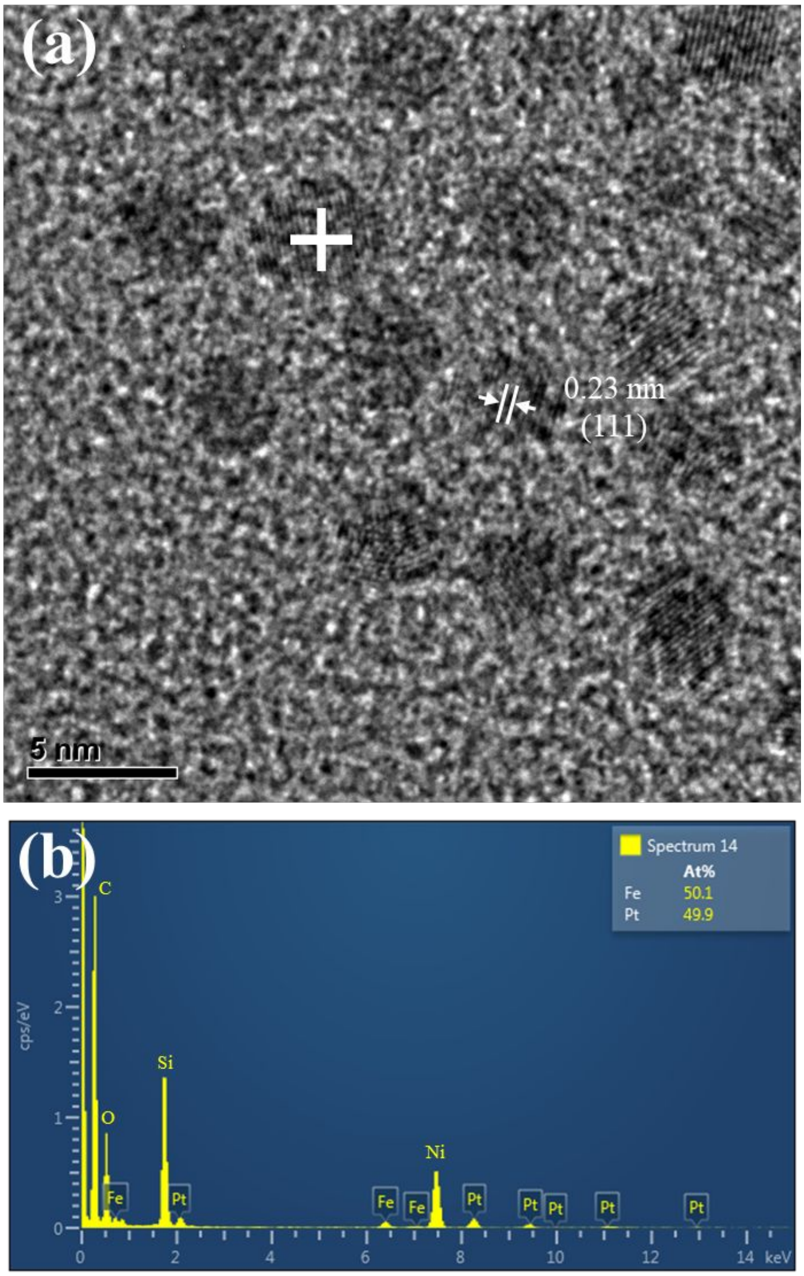

Figure S1 a, HR-TEM image of the FePt nanoparticles and **b,** the corresponding EDS signals of Fe and Pt from white cross as indicated in above (a). The other signals belong to the TEM nickel grid support composed of a carbon and silicon film.

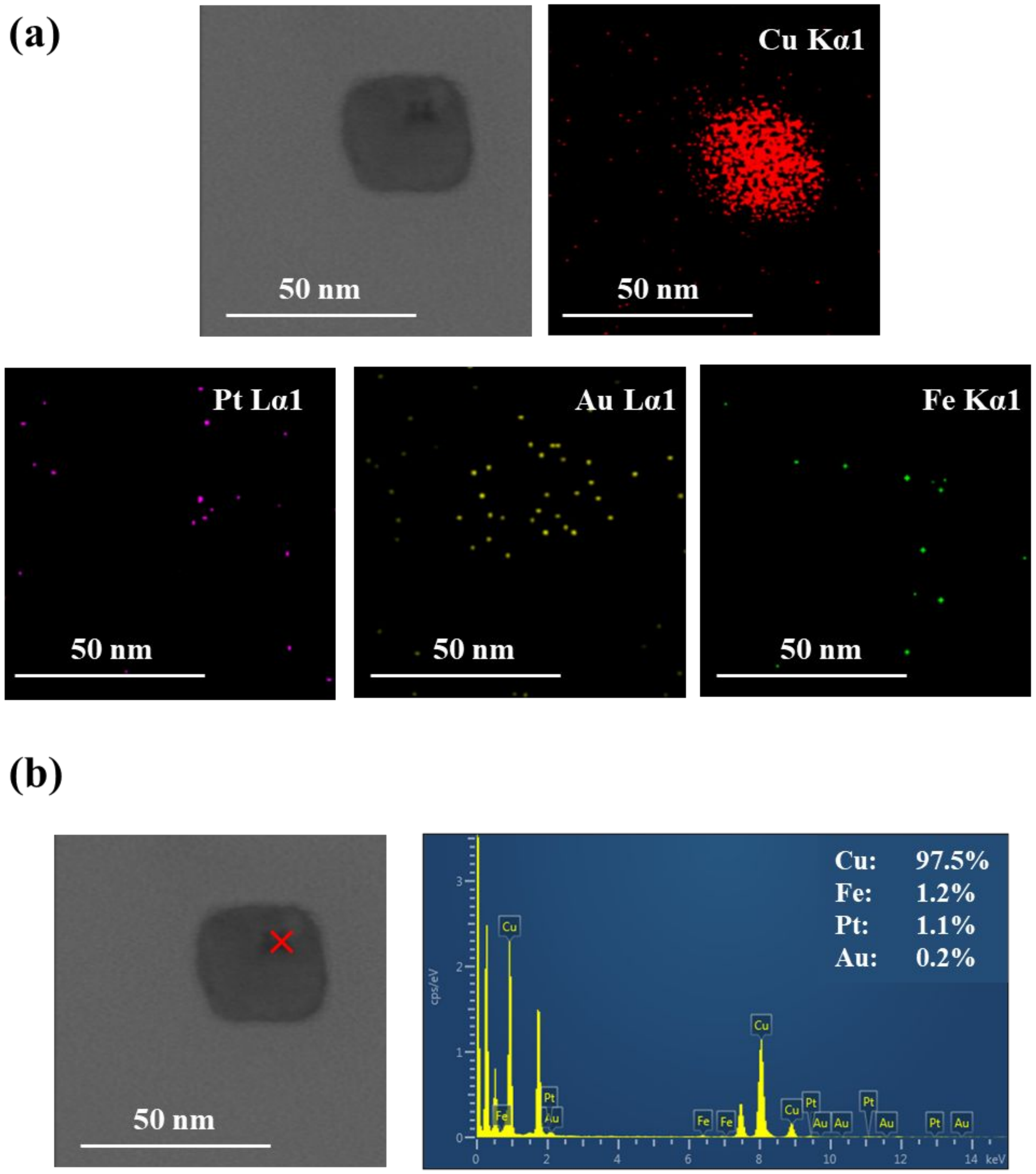

Figure S2 a, HR-TEM image of the single FePt@Cu/Au nanocube and the corresponding EDS mapping of Cu (red), Pt (pink), Au (yellow) and Fe (green). **b,** The corresponding EDS signals of Cu, Fe, Pt and Au from red cross.

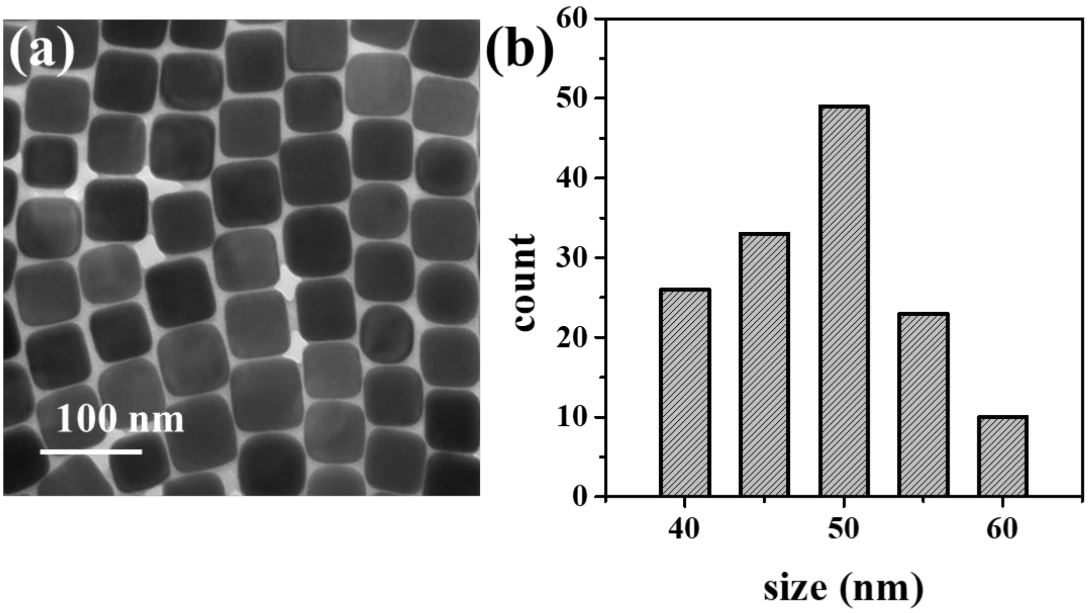

Figure S3. The characteristics of Au nanocubes. **a**, TEM image; **b**, Size distribution in edge length of Au nanocubes.

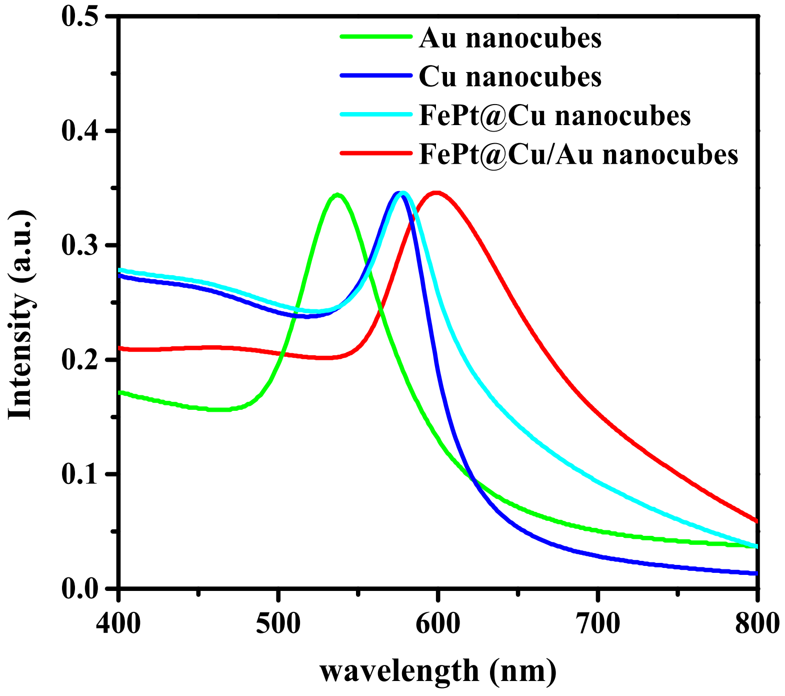

Figure S4. UV-Vis profiles of Au, Cu, FePt@Cu, and FePt@Cu/Au nanocubes.

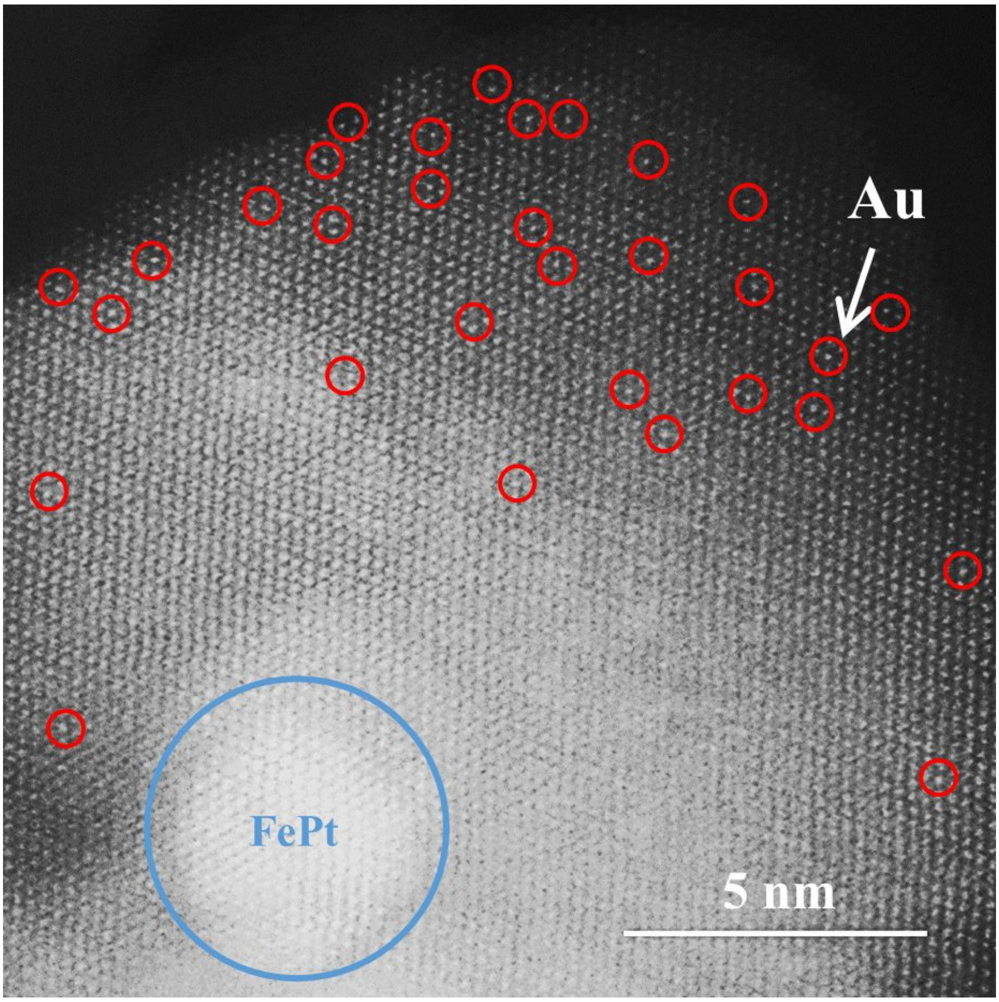

Figure S5. The magnified image of Figure 2a showing high resolution of AC HAADF-STEM image of the FePt@Cu/Au nanocube.

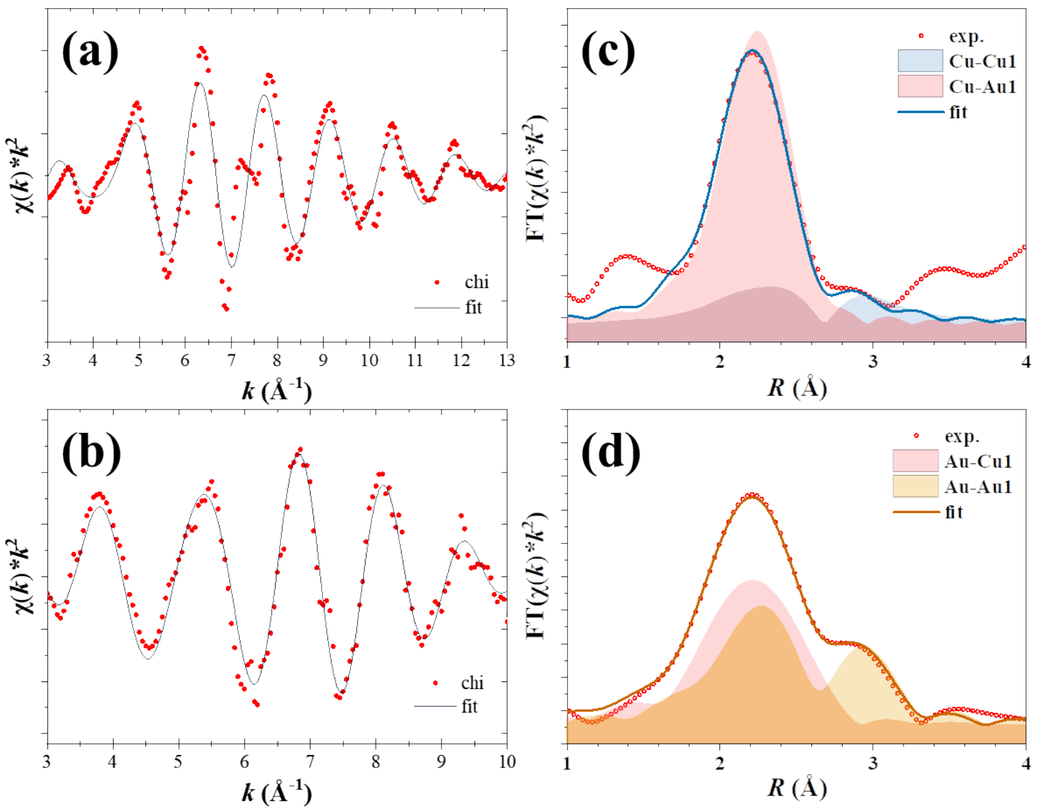

Figure S6 k^2-weighted EXAFS spectra of **a,** Cu K- and **b,** Au L_3-edge; Fourier Transforms at **c,** Cu K- and **d,** Au L_3-edge of FePt@Cu/Au nanocubes.

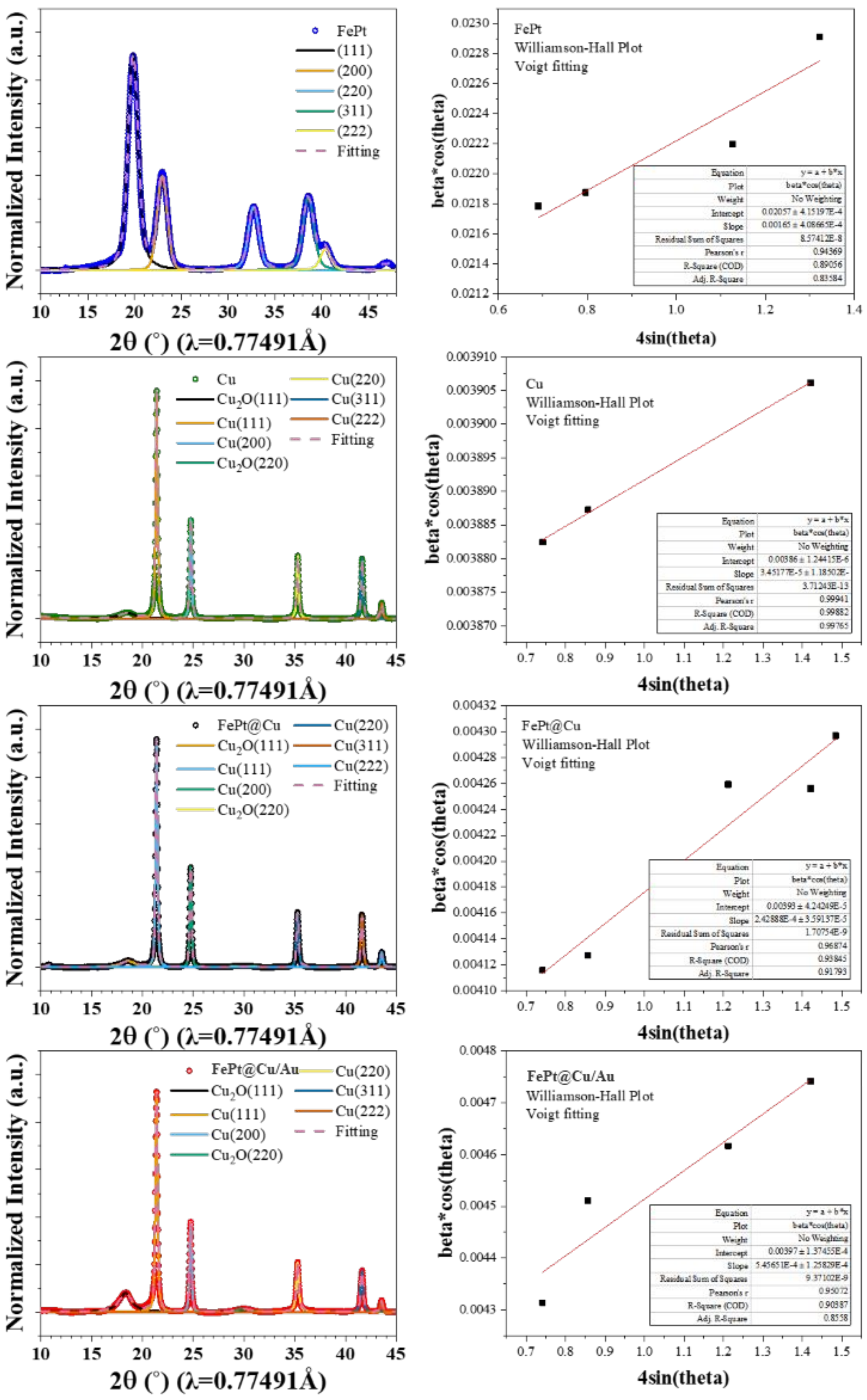

Figure S7 PXRX pattern fitting and Williamson-Hall Plot of sample FePt, Cu, FePt@Cu and FePt@Cu/Au.

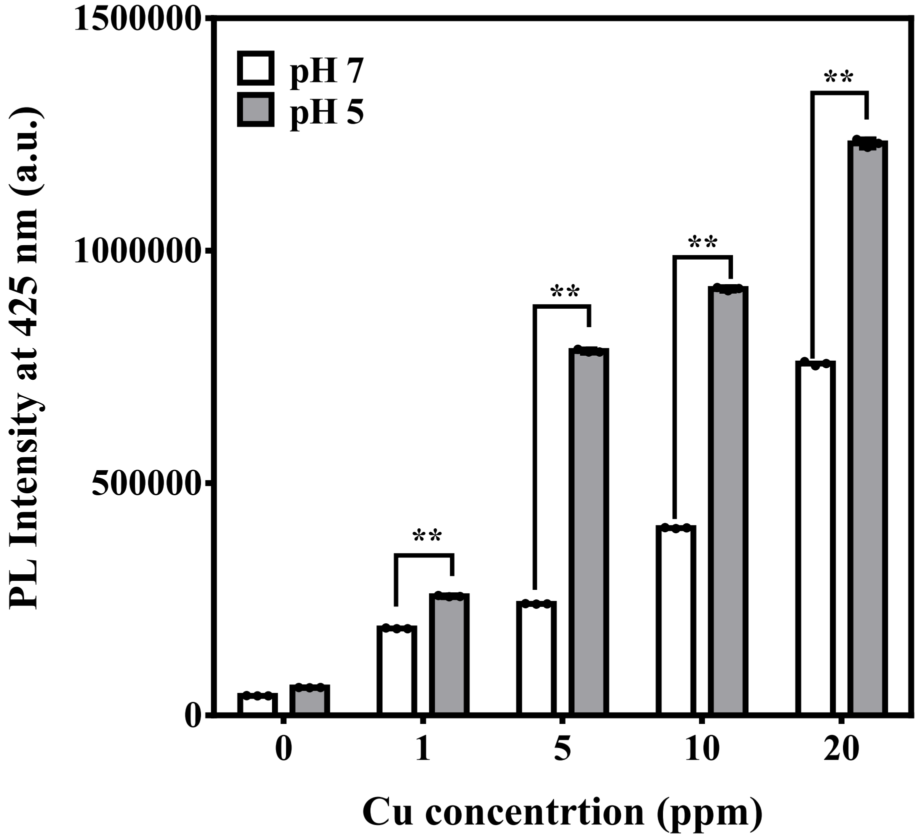

Figure S8 The efficiency of •OH generation at different pH levels (5 and 7) was detected by measuring TPA fluorescence intensity under varying concentrations.

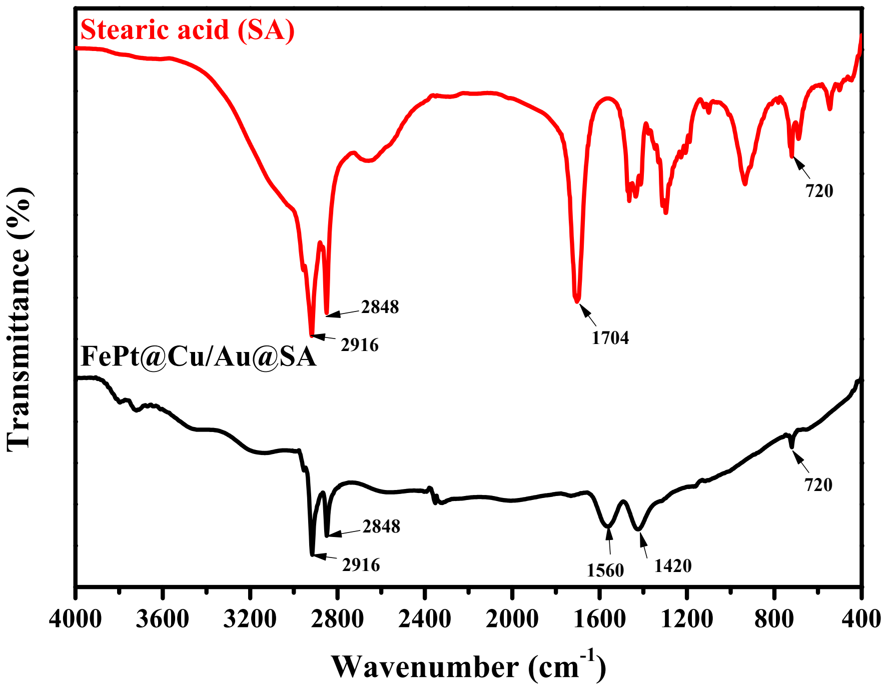

Figure S9 Fourier-transform infrared (FTIR) spectra of FePt@Cu/Au nanocubes with stearic acid (SA) modification.

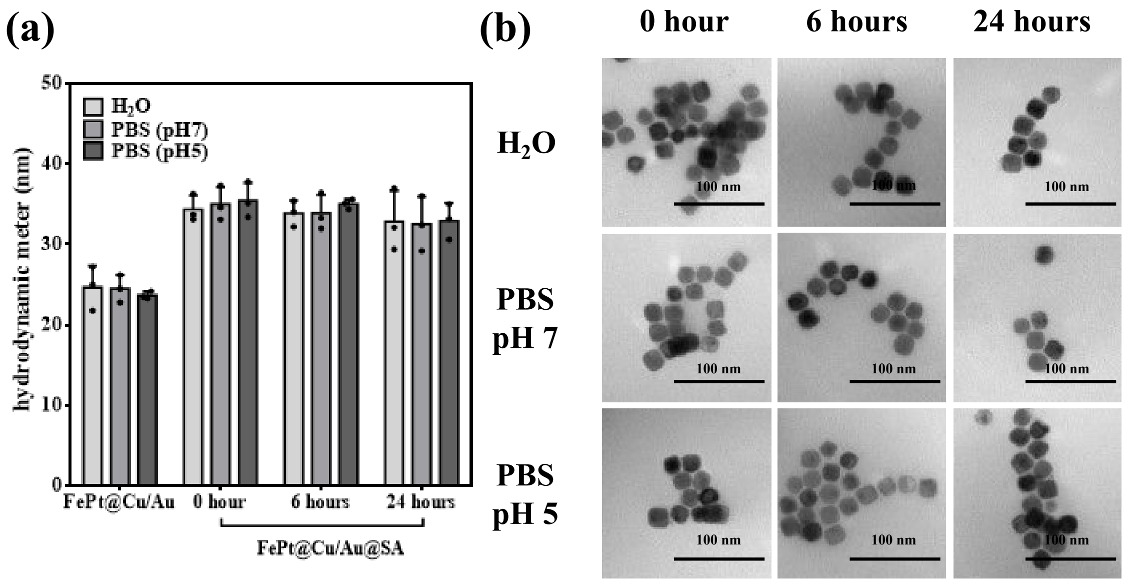

Figure S10 a, DLS size of FePt@Cu/Au and stability of FePt@Cu/Au@SA under different solution conditions. **b,** TEM images revealed the stability of FePt@Cu/Au@SA nanocubes under various solution conditions (H_2O, PBS pH 7, and 5).

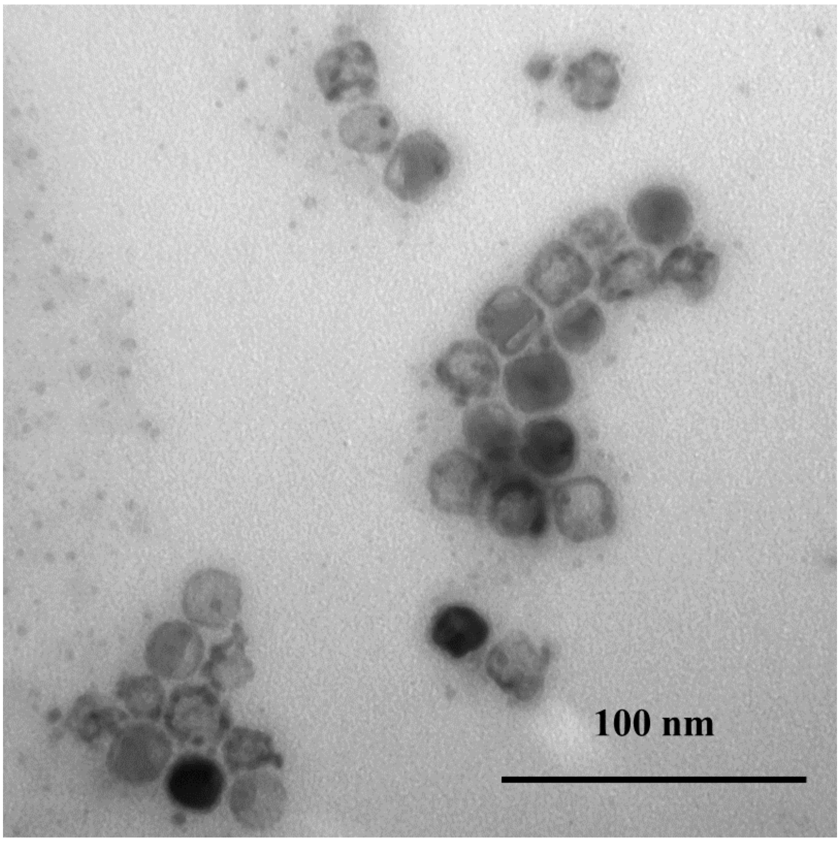

Figure S11 The morphology of FePt@Cu/Au@SA nanocubes treated with HepG2 cancer cells for 24 hours.

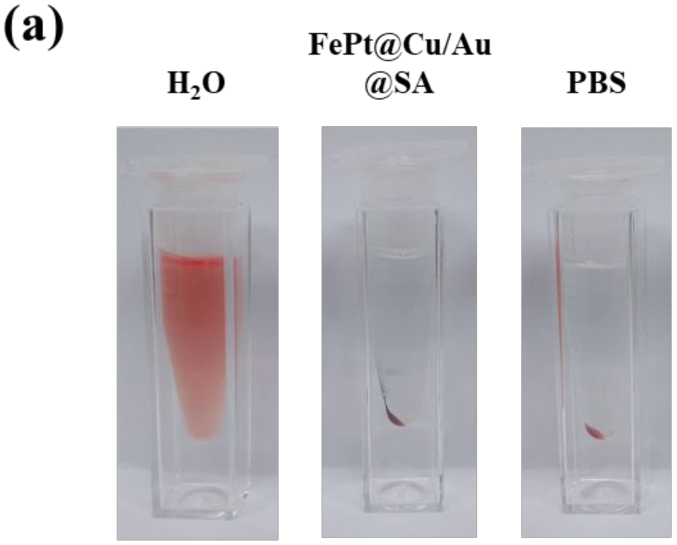

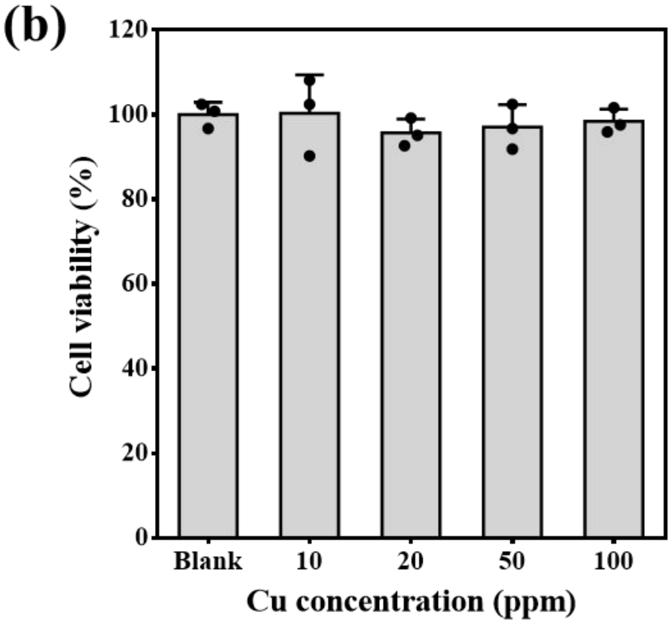

Figure S12 a, Analysis of hemolysis in blood containing 2% red blood cells from FePt@Cu/Au@SA nanocubes. Negative and positive controls conducted by immersing red blood cells in PBS and water, respectively. **b,** Cytotoxicity analysis of vascular endothelial cells treated with FePt@Cu/Au@SA nanocubes. All data were obtained in triplicate.

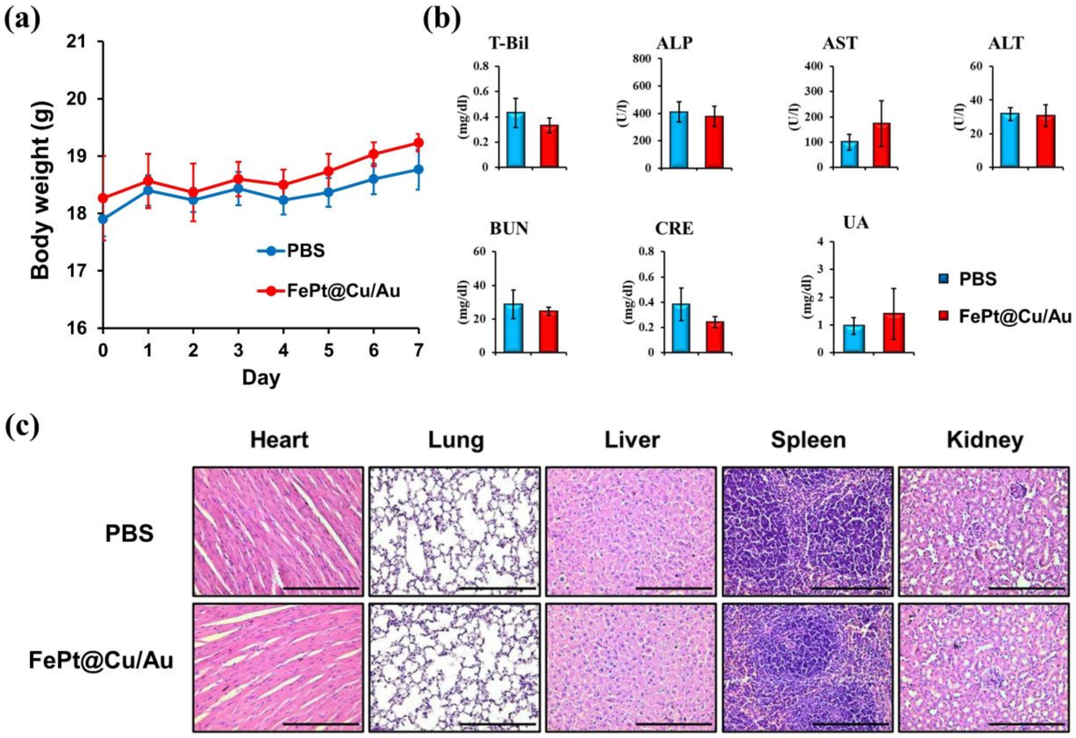

Figure S13 Examining the biosafety of FePt@Cu/Au@SA treatments in C57BL/6 mice. a, Daily monitoring of body weight changes in the PBS-treated group and mice treated with FePt@Cu/Au@SA was conducted for up to 7 days (n=3). **b,** On the 7th day post-injection, blood biochemical analysis was performed on mice (n=3). **c,** Histological morphology of each organ was observed on the 7th day post-injection using H&E staining (Scale bar, 200 μm) (n=3).

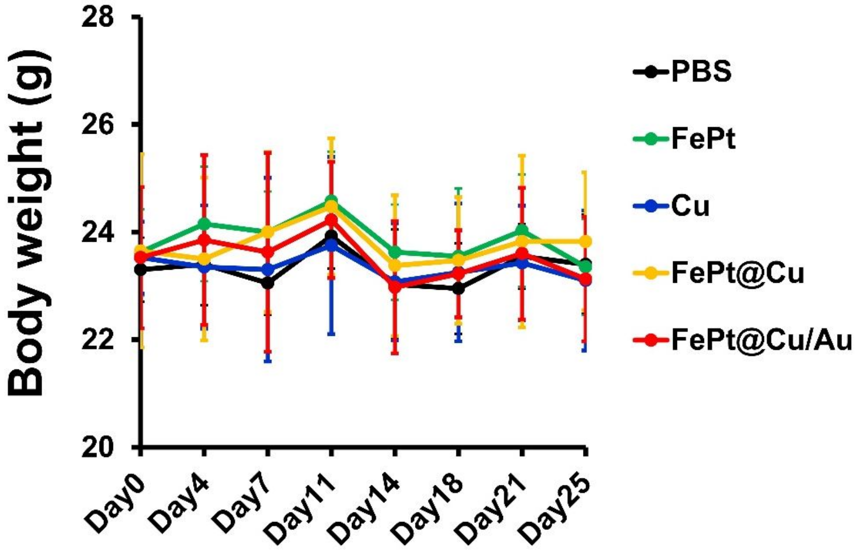

Figure S14 Body weight changes of HepG2-Red-FLuc orthotopic tumor mice in each treatment group (n=4).
